# Increased susceptibility to diet-induced obesity in female mice impairs ovarian steroidogenesis: The role of elevated leptin signalling on nodal activity inhibition in theca cells

**DOI:** 10.1016/j.molmet.2024.102062

**Published:** 2024-11-12

**Authors:** Karolina Wołodko, Tjaša Šentjurc, Edyta Walewska, Elżbieta Laniecka, Magdalena Jura, António Galvão

**Affiliations:** 1 Institute of Animal Reproduction and Food Research of PAS, Department of Reproductive Immunology and Pathology, Olsztyn, Poland; 2 The Royal Veterinary College, University of London, London, NW1 0TU, UK

**Keywords:** Diet induced-obesity, Ovarian steroidogenesis, Nodal, Leptin

## Abstract

**Objectives:**

Susceptibility to obesity in humans is driven by the intricate interplay of genetic, environmental and behavioural factors. Moreover, the mechanisms linking maternal obesity to infertility remain largely understudied. In this study, we investigated how variable susceptibility to obesity in mice affects ovarian steroidogenesis, with a particular focus on the leptin-mediated dysregulation of Nodal signalling pathway in theca cells (TC).

**Methods:**

C56BL/6J (B6) and 129S1/SvlmJ (129) mice, models of maternal obesity (MO), were fed a chow diet (CD) and a high fat diet (HFD) for 16 weeks. To investigate the contrasting effects of leptin on ovarian steroidogenesis, B6 mice pharmacologically treated with leptin for 16 days on CD were used to model hyperleptinemia, while homozygous ob/ob (−/−) mice with genetic leptin deficiency, also on a CD, were used to examine the effects of obesity in the absence of leptin. Following the characterisation of the mouse phenotype, gonadal fat (GON), whole ovaries (WO), ovarian TC and granulosa cell (GC) fractions were collected for mRNA transcription and protein expression analysis. Finally, *in vitro* treated ovarian explants obtained from B6 mice were used to further elucidate the effects of Nodal on steroidogenesis.

**Results:**

The significant gain in body weight (BW) and fat mass (FM) in HFD-fed B6 mice (p < 0.05), was associated with increased mRNA transcription of the adipose tissue expansion genes *Polymerase I and transcript release factor* (*Cavin*), *Secreted frizzled-related protein 5* (*Sfrp5*) and *Mesoderm specific transcript* (*Mest*) in GON (p < 0.05). Furthermore, the HFD-fed B6 mice presented also impaired glucose metabolism and insulin sensitivity (p < 0.05). In contrast, the HFD-fed 129 mice exhibited no changes in BW and FM, maintaining glucose and insulin metabolism. At the ovarian level, decreased protein expression of Steroidogenic Acute Regulatory Protein (StAR) in WO obtained from HFD-fed B6 mice (p = 0.05), was followed by reduced transcription of key steroidogenic genes like *Star* and *Cytochrome P450 17a1* (*Cyp17a*) in TC (p < 0.05). Furthermore, the transcription of *Nodal* and its receptors was downregulated (p < 0.05), whereas mRNA levels of *Suppressor of cytokine signalling 3 (Socs3)* and *SMAD family member 7 (Smad7)* were upregulated in TC obtained from HFD-fed B6 mice (p < 0.05). No changes were seen in the genes regulating steroidogenesis, Nodal signalling, or *Socs3* and *Smad7* activity in the ovaries of HFD-fed 129 mice. Importantly, the pharmacological treatment of lean mice with leptin, upregulated the ovarian transcription of *Socs3* and *Smad7*, while downregulating *Nodal* and its receptors (p < 0.05). Finally, *in vitro* pharmacological inhibition of Nodal signalling pathway in ovarian explants isolated from CD-fed B6 mice decreased the transcription of *Star* and *Cyp17a* in TC (p < 0.05), whereas Nodal treatment of explants obtained from HFD-fed B6 mice restored the transcription of both genes (p < 0.05).

**Conclusions:**

Increased susceptibility to obesity in MO is associated with systemic hyperleptinemia and hypoestrogenism due to compromised ovarian steroidogenesis, largely driven by the inhibitory effects of leptin-Smad7 pathway on Nodal signalling activity in the TC compartment of ovarian follicles.

## Highlights

• Increased susceptibility to obesity in mice is linked to compromised ovarian steroidogenesis.
• The expression of steroidogenic enzymes in ovarian theca compartment is decreased in obese mice.
• Leptin-mediated upregulation of Smad7 suppresses Nodal activity in theca cell from obese mice.
• Nodal signalling supports ovarian steroidogenesis.

## 1 Introduction

Susceptibility to obesity and body weight (BW) gain arises from a multitude of factors, including dietary habits, lifestyle, gender and energy balance [1]. Importantly, individual metabolic profile regulates lipid metabolism, fat distribution, triglyceride clearance, and cholesterol homeostasis [1]. Mouse models with different susceptibility to obesity have proven instrumental to our understanding of the mechanisms underlying the response to obesogenic stimuli and the occurrence of obesity-related comorbidities [2]. The C57BL/6J (B6) mouse strain presents rapid gains in body weight, often associated with the establishment of insulin and leptin resistance when exposed to a high fat diet (HFD), being therefore frequently classified as obesity prone (OP) [3,4]. In contrast, the 129S1/SvlmJ (129) mouse strain presents moderate gains in BW following HFD treatment, despite the lower levels of spontaneous locomotor activity [2,5], which defines the strain as obesity resistant (OR). Such variability in the response to HFD treatment conveniently portrays different susceptibility to obesity in humans. Furthermore, they represent an invaluable model for studying the impact of maternal obesity on reproductive function, providing insights into the complex interaction between metabolic health and fertility.

Obesity is globally prevalent and presents a myriad of associated comorbidities. Importantly, the relationship between obesity and infertility remains largely understudied. Obese women tend to show menstrual abnormalities, decreased conception rates, and poor assisted reproductive technology (ART) outcomes [6]. Obesity is also associated with hormonal dysregulation, characterised by altered serum estrogen levels [7] and metabolism [8] and decreased levels of sex hormone binding globulin [9]. The endocrine imbalance observed in obese mothers is known to dysregulate the activity of the hypothalamic–pituitary–gonadal axis, leading to increased ovarian androgen production [10]. Moreover, the expansion of gonadal fat pad (GON) in obese mothers is a source of inflammatory mediators and adipokines, which were shown to compromise ovarian function and fertility [11]. Increased lipid content has been documented in the follicular fluid of obese women [12] and the occurrence of lipotoxic events has been observed in ovarian cells from obese female mice [13], contributing to the pathogenesis of ovarian failure in obese mothers. Such events often result in impaired oocyte growth and maturation [13], leading to pregnancy complications and decreased live birth rates [14]. These outcomes reflect the significant decline in reproductive performance among obese mothers.

The ovarian follicle harbours the oocyte, nurturing its development and maturation, a role mainly accomplished by the activity of the somatic granulosa (GC) and theca cells (TC) [15,16]. Research in both obese women and mouse models of maternal obesity has revealed functional changes in ovarian TC - the primary ovarian steroidogenic component, and in GC, the oocyte companions known to control its maturation and developmental competence [17]. Cholesterol, the primary precursor for steroidogenesis, can either be synthesised within steroidogenic cells or taken up from circulating lipoproteins, a process hormonally regulated [18]. In mammals, lipid droplets play a crucial role in lipid homeostasis by storing cholesterol esters, which are later used for steroid hormone production [19]. When cholesterol is not synthesised internally, it is obtained through the hydrolysis of these cholesterol esters stored in lipid droplets [18]. Perilipin (PLIN1), a well-established marker of lipid droplets in TC, regulates lipolysis, and the generation of the necessary precursors for steroid synthesis [18,19]. According to the two-cell theory, estrogen production entails a multi-step process, whereby androgens are initially synthesised in TC through the activity of the steroidogenic acute regulatory protein (StAR), cytochrome P450 family 11 subfamily A member 1 (CYP11A1), CYP17A1, 3b-Hydroxysteroid dehydrogenase (3bHSD), and 17bHSD [20]. Subsequently, testosterone is transported to the GC and converted into estrogen by CYP19A1 [21]. Steroidogenesis is initiated by transcription factors, like *Nuclear receptor subfamily 5, group A, member 1* (*Nr5a1)* [22,23] or *GATA binding protein 4* (*Gata4)* [23,24], known to control the expression of StAR and 3bHSD, or by hormones like luteinising hormone (LH) [25]. As a results, estradiol (E2) is synthesised, further governing oocyte maturation [26] and proliferation of the uterine endometrium [27], among others. Literature has shown the dysregulation of steroidogenesis in obese mice through the upregulation of *Cyp17a1* in TC and increased testosterone production [28]. However, other reports described the leptin-mediated dysregulation of steroidogenesis and decreased E2 levels in rats treated with a cafeteria diet [29] or compromised steroidogenesis and decreased E2 production in male obese mice [30]. Conflicting observations highlight the complexity of steroidogenic regulation and the need for further research elucidating the mechanisms underlying impaired steroidogenesis in obese women.

Leptin is known as an established modulator of reproductive function, acting both centrally and peripherally. In addition to its well-documented role regulating the neuroendocrine reproductive axis [31], leptin is also known to act at the ovarian level, governing folliculogenesis [32], steroidogenesis [33] and ovulation [34]. The expression of leptin and its receptors has been reported in various ovarian components, like TC, GC and oocytes across multiple species [[35], [36], [37]]. Notably, our research has revealed the establishment of leptin resistance in the ovaries of diet-induced obese (DIO) mice, largely mediated by increased expression of suppressor of cytokine signalling 3 (SOCS3) [3]. Another study in human GCs unveiled the inhibitory effects of leptin on the cyclic adenosine monophosphate (cAMP)-stimulated expression of StAR protein [38]. In summary, leptin closely regulates steroidogenesis, and the development of leptin resistance in the ovaries of obese mothers is expected to negatively affect steroidogenic activity in both TC and GC compartments.

The Transforming growth factor beta (TGFb) superfamily represents a conserved group of cytokines implicated in the regulation of ovarian function [39]. Numerous studies demonstrated the presence of both TGFb and Nodal signalling components in the ovaries of various species, including humans [40], horses [41] and mice [42]. Nodal has long been known for its pivotal role in vertebrate embryonic development [43]. However, recent research on zebrafish has shed light on Nodal activity controlling follicular growth, steroidogenesis, and oocyte maturation [44]. Noteworthy, the expression of Nodal and its receptor Activin receptor-like kinase 7 (Alk7) was shown in both TC and GC of rat ovarian antral follicles [45] and largely implicated in the initiation of apoptosis during follicular atresia [42]. Another TGFb superfamily ligand, TGFb1, was shown to modulate the secretory activity of equine luteal cells *in vitro* [46]. Importantly, TGFb1 treatment of human GC downregulated StAR expression and reduced progesterone production *in vitro* [47]. Therefore, we presently investigate the involvement of Nodal signalling in ovarian steroidogenesis modulation in obese mice.

We hypothesise that impaired ovarian steroidogenesis in obese mothers results from the concurrent dysregulation of leptin and Nodal signalling pathways, which together compromise steroidogenic activity within the TC compartment. We found that HFD-fed B6 mice displayed greater body weight and endocrine imbalance characterised by systemic hyperleptinemia and hypoestrogenism, accompanied by ovarian leptin resistance compromised steroidogenesis. Moreover, *ex vivo* studies with ovarian explants established the link between decreased Nodal signalling activity and reduced steroidogenesis in TC from obese mice.

## 2 Materials and methods

### 2.1 Animal protocol

Breeding pairs of B6, 129, and B6. Cg-Lepob/J (ob/ob) mouse strains were obtained from The Jackson Laboratory (Bar Harbor, Maine, USA). At 8 weeks (wk) of age, B6 and 129 mice were randomly assigned to two groups (n = 12 per group). The control group was fed an ad libitum chow diet (CD, 11% energy from fat, 5053 rodent diet 20, LabDiet IPS, London, UK), while the experimental group was placed on a HFD (58% energy from fat, AIN-76A 9G03, LabDiet IPS) for 16 wk. Food intake (FI) was measured weekly. For phenotype characterisation, body composition was monitored using nuclear magnetic resonance (NMR, Bruker, Rheinstetten, Germany). BW, fat mass (FM), lean mass (LM) and adiposity index (AI, fat mass/lean mass) were measured at the end of the protocol. Vaginal cytology was conducted during the last twelve consecutive days of the protocol. Vaginal lavage was performed using saline, and the collected smears were placed on slides and stained with the Diff-Quick Staining Set (Diff- Quick Color Kit, Rapid Staining Set, Medion Grifols Diagnostics AG, Duedingen, Switzerland) for cell identification [48]. The pharmacological hyperleptinemic (LEPT) protocol consisted of 10 wk old B6 mice fed a CD and injected intraperitoneally with saline (C) or leptin (L) for 16 days, as previously described [3]. Heterozygous (+/?) and homozygous (−/−) ob/ob mice with genetic deficiency of leptin were fed a CD and culled at 12 wk of age.

### 2.2 Superovulation protocol and MII oocyte collection

Mice in estrus were superovulated (SO) for cumulus–oocyte complex (COC) collection from the oviductal ampulla. Metaphase II (MII) oocytes were isolated, and ovaries were harvested for further analysis. For the SO of B6, mice were intraperitoneally injected with pregnant mare's serum gonadotropin (PMSG, G4877, 5IU, Sigma Aldrich, Saint Louis, Missouri, USA) followed by the administration of human chorionic gonadotropin (hCG, Chorulon, 5IU, MSD Animal Health, Boxmeer, Holland) 48 h later. In 129 mice, the same hormonal treatment was given, but with a 72 h (h) interval between PMSG and hCG injections. Subsequently, 18 h after hCG injection the animals were sacrificed, the ovaries collected and COC retrieved from the oviduct, followed by MII oocyte counting and *in vitro* fertilisation (IVF) for embryo generation.

### 2.3 Glucose and insulin tolerance tests

In both tests, the animals were fasted for 6 h. In the glucose tolerance test (GTT), mice were injected intraperitoneally with 20% glucose (2 g/kg body weight; G7528, Sigma Aldrich). In the insulin tolerance test (ITT), mice were injected with insulin (0.75 IU/kg body weight; #256A01; Caninsulin, Intervet). Blood glucose levels were measured with a blood glucose monitor (ACCU-CHEK) prior to the initial injection and then at 10, 20, 40, 60, 80, 100 and 120 min post-injection [49].

### 2.4 Tissue collection

Mice in estrus were sedated [50], and blood was collected by cardiac puncture for enzyme-linked immunosorbent assay (ELISA) analysis. After sacrificing the mice by cervical dislocation, GON was collected, rinsed with phosphate buffered saline (PBS, 0.1M, pH = 7.4), and transferred to TRI Reagent for mRNA isolation. Ovaries were isolated from the genitalia and transferred to either TRI Reagent (T9424, Sigma Aldrich) for mRNA isolation, or radioimmunoprecipitation assay buffer (RIPA, 89901, ThermoFisher Scientific) supplemented with a protease inhibitor cocktail (PIC, P8340, Sigma Aldrich), phenylmethylsulfonyl fluoride (PMSF, P7626, Sigma Aldrich) and phosphatase inhibitor (88667, Thermo Fisher Scientific) for protein analysis. For staining, ovaries were fixed in 4% paraformaldehyde (PFA) for further haematoxylin and eosin or immunofluorescent (IF) staining. Samples were frozen and stored at −80 °C for further analysis.

### 2.5 Ovarian cells and GV oocyte collection

Immediately after euthanasia, the ovaries from superovulated (SO) animals were transferred to M2 medium (M7167, Sigma Aldrich). The germinal vesicle (GV) oocytes were harvested by puncturing ovarian follicles with a 16 G needle. The released GV oocytes were collected, washed in M2 medium, and counted. The remaining ovarian tissue was washed in PBS, transferred to TRI Reagent, and collected as the TC, then stored at −80 °C. GC were released during follicle puncture and denuding of GV oocytes using a mouth pipette fitted with a glass needle [56]. The GC suspension was centrifuged (600 g, 3 min, 4 °C), washed in PBS, the pellet resuspended in TRI Reagent and frozen at −80 °C. The expression of cell-specific markers for each population was subsequently measured in both fractions [51].

### 2.6 In vitro fertilisation and embryo culture

Embryos were generated via IVF. Sperm activation, fertilisation and embryo culture were performed in droplets of medium cultured under paraffin oil. Males were sacrificed and the sperm was collected from the epidydimal tail and transferred to Fertiup (KYD-005-EX, CosmoBio) for capacitation at 37 °C, 5% CO_2_ for 60 min. Within 3 min (min) of sacrificing the females, the COC was collected and transferred to CARD medium (KYD-005-EX, CosmoBio). Subsequently, 5–10 μl of sperm was added to the COC suspension. After 3 h of incubation, zygotes were transferred to embryo culture medium (Continuous Single Culture Complete with HSA; 90165-2x20ML; Irvine Scientific) and cultured for 3.5 days. At 24 h post-fertilisation, the number of 2-cell embryos was assessed, and blastocysts were counted 3.5 days after fertilisation.

### 2.7 In vitro culture of ovarian explants

Superovulated mice were euthanised, and ovaries collected in M2 medium before being quartered with a scalpel blade. The ovarian explants were then washed in PBS and transferred to a 24-well plate (3526, Corning) containing 1 ml of DMEM/F12 medium (21041025, Gibco) supplemented with 5% fetal bovine serum (FBS, F6765, Sigma Aldrich) and 1% antibiotics (A5955, Sigma Aldrich). The explants were incubated at 37 °C in 5% CO2 and 100% humidity for 3 h to equilibrate. Afterwards, the medium was replaced with DMEM/F12 supplemented with 0.1% bovine serum albumin (BSA, A9418, Sigma Aldrich) and pre-incubated for 1 h prior to the final treatment. To control for maternal effects, ovarian explants from different mice were randomly pooled and co-cultured. Each ovary was divided into four equal parts, and four ovarian quarters from separate females were combined to form a single sample (n = 1). The explants were treated with either 500 or 1000 ng/ml of Nodal (1315-ND, R&D Systems) or 0.1, 1 or 5 μM of a selective inhibitor of the Activin receptor type 1 B (Alk4), TGFb type I receptor (Alk5) and Alk7, SB431542 (SB, S4317, Sigma Aldrich). The treatment doses used in the *in vitro* experiments were selected based on prior studies examining the effects of Nodal [[58], [59], [60]] and SB [[61], [62], [63]] on ovarian cells. Ovaries collected from CD-fed B6 mice were treated with SB, while ovaries collected from HFD-fed B6 mice were treated with Nodal. Samples were cultured for 12 h for mRNA isolation and 24 h for protein extraction. Saline was used as the control for Nodal treatment, whereas dimethyl sulfoxide (DMSO) was the control for SB. After incubation, the ovarian explants were punctured and TC collected.

### 2.8 MTS cytotoxicity assay

Ovarian explants were cultured in 24-well plates for 24 h. Subsequently, ovarian cell viability was determined using the CellTiter 96® AQueous Non-Radioactive Cell Proliferation Assay (G3582, Promega), according to the manufacturer's instructions. Four h before the end of the treatment, 0.2 ml of 3-(4,5-dimethylthiazol-2-yl)-5 (3-carboxymethoxyphenyl)-2-(4-sulfophenyl)-2H-tetrazolium (MTS) solution was added to each well. Following an additional 4 h incubation, absorbance was measured at 490 nm using the BioTek Synergy H1 Multimode Reader. Cell viability was calculated relative to the control group for each experimental condition.

### 2.9 RNA isolation and cDNA synthesis

Adipose tissue, WO, TC and GC fractions were collected (n = 8/group) for mRNA isolation and homogenized in TRI Reagent according to the manufacturer's instructions (Molecular Research Centre). The suspension was vigorously mixed and incubated for 5 min at room temperature (RT). After centrifugation (9400 g, 4 °C, 15 min), the supernatant was transferred to a new tube and thoroughly mixed with 100 μl of 1-bromo-3-chloropropan (BCP, BP151, Molecular Research Centre), followed by a 10 min incubation at RT. Samples were centrifuged again (13500 g, 4 °C, 15 min), and the aqueous phase was transferred to a new tube, mixed with an equal volume of isopropanol and incubated at −80 °C for 60 min. The RNA was pelleted by centrifugation (20,000 g, 4 °C, 15 min), washed three times with 75% ethanol, and incubated overnight at −80 °C. The following day, samples were centrifuged (20,000 g, 4 °C, 15 min), the RNA pellet was dried and resuspended in 20 μl of RNase-free water (W4502, Sigma Aldrich) supplemented with RNase Inhibitor (RiboProtect, RT35, BLIRT). RNA quality and concentration were assessed using NanoDrop1000 spectrophotometer, with absorbance ratios at 230, 260, and 280 nm (A260/A280 and A260/A230) confirming the quality and concentration of the extracted mRNA. A total of 1 μg of RNA was used for reverse transcription and cDNA synthesis using the Maxima First Strand cDNA Synthesis Kit for real-time polymerase chain reaction (PCR) (K1642, ThermoScientific) according to the manufacturer's instructions. The cDNA was stored at −20 °C until real-time PCR.

### 2.10 Real-time polymerase chain reaction

Real-time PCR was conducted using the 7900 Real-Time PCR System (Applied Biosystems, Warrington, UK) with either Maxima SYBR Green/ROX qPCR Master Mix (K0223, Thermo Scientific) or RNA-TO-CT 1-step TaqMan Master Mix (43929382, Thermo Scientific). Primers for SYBR Green Master Mix analysis were designed using Primer 3.0 v.0.4.0. software [51], based on gene sequences from GeneBank (NCBI), as described previously [46]. Details of the primer sequence, PCR product length, and GeneBank accession numbers are presented in Supplementary Table 1. Primers and probe sequences used for TaqMan PCR are presented in Supplementary Table 2. The reaction mixture consisted of 4 μl of cDNA (50 ng), 1 μl of forward and reverse primers (at 80 nM or 160 nM), and 6 μl of SYBR Green PCR Master Mix, bringing the total reaction volume to 12 μl. Real-time PCR was carried out as follows: an initial denaturation step at 95 °C for 10 min, followed by 45 cycles of denaturation at 95 °C for 15 s and annealing at 60 °C for 1 min. After each PCR run, melting curves were generated to confirm the amplification of a single product. Both the target gene and a housekeeping gene (HKG) - *Ribosomal protein L37* (*Rpl37*, primers in Supplementary Table 1) or *Eukaryotic translation initiation factor 5a* (*Eif5a)* were run simultaneously, and reactions were performed in duplicate wells in a 384-well optical reaction plate (4306737, Applied Biosystems). The validation of the HKG was made with NormFinder for each experimental group. Real-time PCR results were analysed with the Real-time PCR Miner algorithm [52].

### 2.11 Protein isolation and western blotting analysis

Protein expression in mouse ovaries was assessed by western blotting (WB). Freshly collected tissues were lysed in RIPA buffer with protease and phosphatase inhibitors and mechanically homogenised with a lancet, as previously described [3]. The lysates were incubated for 1 h on ice. Subsequently, the samples were centrifuged (20000 g, 4 °C, 15 min), and the supernatant collected and stored at −80 °C until further analysis. Protein concentration was determined using the copper/bicinchoninic acid assay (Copper (II) Sulfate, C2284; Sigma and Bicinchoninic Acid Solution, B9643; Sigma Aldrich) [53]. WB was performed as previously described [3]. Briefly, after electrophoresis on 4–15% acrylamide gel (4561086, BioRad), proteins were transferred to a polyvinylidene difluoride (PVDF) membrane. Membranes were incubated for 1 h in blocking solution (5% BSA; A2153, Sigma Aldrich) or 5% skimmed milk, followed by overnight incubation with the primary antibody at 4 °C. The blots were incubated with the following primary antibodies: anti-LH receptor (LHR, sc-26341, Santa Cruz Biotechnology), anti-phospho-SMAD2/3 (8828, Cell Signalling), anti- SMAD2/3 (8685, Cell Signalling), anti-StAR (ab96637, Abcam), anti-SOCS3 (sc73045, Santa Cruz Technologies) and anti B-actin (A2228; Sigma Aldrich). After washing, membranes were incubated with the corresponding secondary antibody: goat polyclonal (GP) anti-rabbit alkaline phosphatase-conjugated antibody (1:30000, A3687, Sigma Aldrich), GP anti-rabbit horse radish peroxide-conjugated antibody (1:20000, 31460, Thermo Fisher Scientific), rabbit polyclonal anti-goat alkaline phosphatase-conjugated antibody (1:10000, A7650, Sigma Aldrich). Detection was performed using chromogenic substrate NBT/BCIP diluted 1:50 (11681451001; Roche) in alkaline phosphate buffer or SuperSignal™ West Femto Maximum Sensitivity Substrate (34096, Thermo Scientific). The blots were then scanned using the ChemiDoc Imaging System (BioRad, Hercules, California, USA) and the specific bands were quantified using ImageLab Software (BioRad). Finally, the band density for each protein was normalised against B-actin (1:10000, A2228, Sigma–Aldrich).

### 2.12 Immunostainings

Ovaries collected from SO mice (n = 3/group) were fixed in 4% PFA (28908, Pierce) at 4 °C for 24 h. Following fixation, the samples were washed and dehydrated in 70% ethanol before being embedded in paraffin. The paraffin-embedded tissues were then sectioned into 5 μm slices using a microtome. The sections were treated with xylene for deparaffinisation and subsequently rehydrated through a series of ethanol solutions. For immunohistochemical staining, the sections were stained with Mayer's Haematoxylin Solution for 7 min (MHS16, Sigma Aldrich), followed by staining with Eosin Y Aqueous Solution for 30 s (HT110216, Sigma Aldrich). Finally, the sections were mounted in DPX medium (255258.1610, POCH). Ovarian morphology was assessed using Axio Observer Systems Z1 microscope (Carl Zeiss Microscopy GmbH). For immunofluorescence staining, the rehydrated slices were permeabilised by performing two consecutive 5 min incubations in 0.1% Tween 20 (P7949, Sigma Aldrich) diluted in phosphate-buffered saline (PBST). Subsequently, the slides were boiled in citrate buffer at 90 °C for 40 min, then cooled and washed in PBS. The sections were then incubated in Fish Serum Blocking Buffer (37527, Thermo Fisher) for 2 h at RT, followed by incubation with Sudan Black (199664, Sigma Aldrich) for 30 min. The sections were incubated with anti-perilipin 1 antibody solution (ab3526, Abcam, 1:1000) or polyclonal rabbit IgG (ab37415, Abcam, 1:5000) overnight at 4 °C. The following day, the sections were thoroughly washed in PBST for 60 min, followed by a 2 h incubation at RT with the secondary antibody (donkey anti-rabbit - A21207, Thermo Fisher, 1:1000). After another round of washing, the sections were incubated with 4′,6-Diamidine-2-phenylindole dihydrochloride (DAPI) solution (10236276001, Roche) for 30 min at RT. They were washed in PBST once more and finally mounted in Slow Fade Mounting Medium (S36937, Invitrogen) and stored at 4 °C until imaging. Images were captured using 40x/1.2A or 63x/1.4A oil immersion objectives on a LSM800 confocal microscope (Carl Zeiss, Germany). The quantification of immunofluorescent staining intensity was done with Zeiss ZEN 3.7 software, and involved the following steps: i) individual antral follicles were identified based on morphology and the theca cell layer outlined for quantification; ii) the granulosa cells were subsequently outlined and their area quantified, serving as a control region for background intensity; iii) the intensity in theca cells was measured above the minimum threshold, after removing the background intensity. Results represent the average intensity across antral follicles in three ovarian sections from different mice per treatment.

### 2.13 ELISA

Mice were sacrificed (n = 8/group), and blood was collected after cardiac puncture. The samples were centrifuged (180 g, 4 °C, 10 min), and the plasma was collected and stored at −80 °C. Levels of plasma leptin were assessed with a commercial ELISA kit (90030; Crystal Chem), while E2 was measured with Affi ELISA (AFG-SM-04406; AffiGen), following the manufacturer's instructions. The intra- and interassay coefficient of variation (CVs) for the leptin ELISA kit were <10%.

### 2.14 Statistical analysis

Statistical analysis was performed using GraphPad Prism 10.0. The D'Agostino-Pearson omnibus normality test was conducted followed by the nonparametric Mann–Whitney test. Food intake results were analysed using multiple t-tests. Data are presented as the mean ± standard deviation (SD) of three or more independent replicates. Significance was defined as p < 0.05.

### 2.15 Statement of ethics

All experiments were approved by the Local Committee for the Ethical Treatment of Experimental Animals of Warmia-Mazury University (Agreement No. 81/2015; 14/2018), Olsztyn, Poland and were performed according to the Guide for the Care and Use of Laboratory Animals, as endorsed by European legislation.

## 3 Results

### 3.1 Increased gain in body weight and fat mass in obesity prone mice is associated with impaired glucose metabolism and altered leptin plasma levels

**Figure 1:**
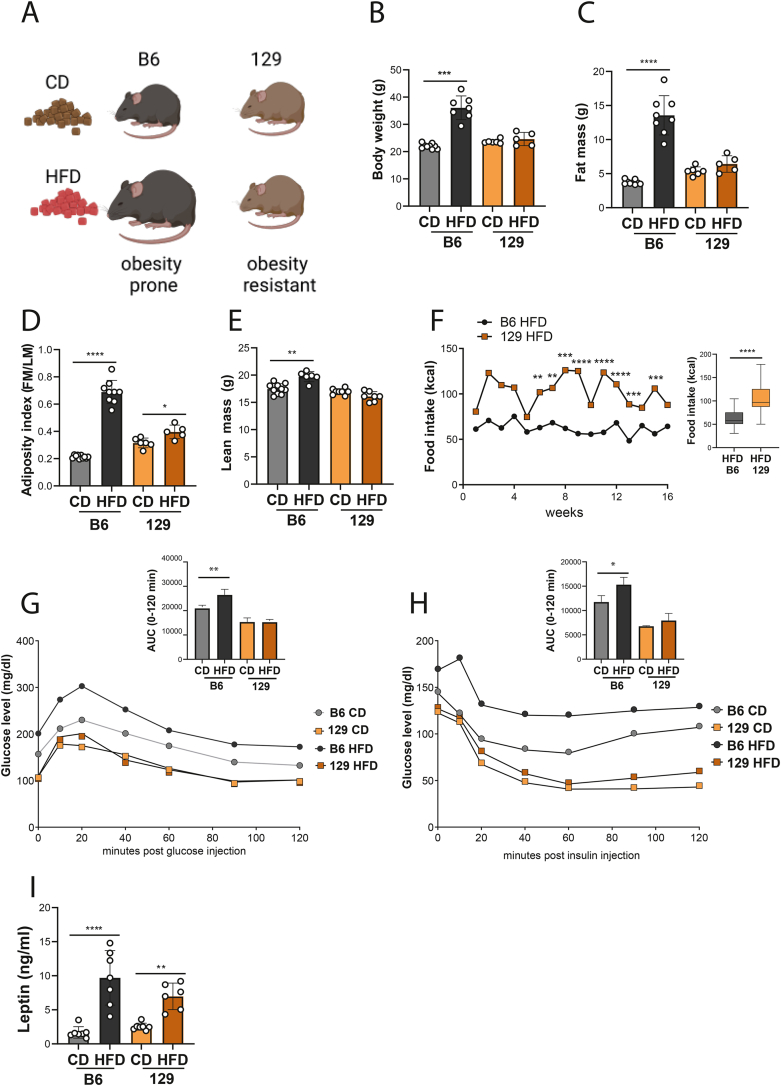
**Obesity prone B6 mice, but not obesity resistant 129, present body weight gain, fat accumulation, and impaired glucose metabolism and insulin sensitivity after diet induced obesity.** (A) Experimental design: obesity prone (C57BL/6J, B6) and obesity resistant (129S1/SvlmJ, 129) mice were fed either chow diet (CD) or high fat diet (HFD) for 16 weeks. Phenotype characterisation included the measurement of (B) body weight (BW), (C) fat mass (FM), (D) adiposity index (AI, FM/lean mass [LM]) and (E) LM. (F) Food intake (FI) presented as weekly caloric intake; the dot plot represents mean caloric intake throughout the experiment; the box whisker plot shows the average caloric intake during the experiment in HFD groups from B6 and 129. Blood glucose levels were measured within 120 min after challenging the animals with glucose in the (G) glucose tolerance test; and insulin in the (H) insulin tolerance test. Bar plots represent the area under the curve was calculated for each group (results presented as mean ± standard deviation). (I) Plasma leptin level measured by enzyme linked immunosorbent assay (ELISA). The differences between groups were analysed using Mann–Whitney test. N = 7–8. Grey bars- B6 CD group, black bars- B6 HFD group, orange bars- 129 CD group, brown bars- 129 HFD group. Asterisks indicate significant differences: ∗p < 0.05; ∗∗p < 0.01; ∗∗∗p < 0.001; ∗∗∗∗p < 0.0001. Diagrams generated with BioRender.com. (For interpretation of the references to color in this figure legend, the reader is referred to the Web version of this article.)

In order to characterise phenotypic response to HFD treatment in mouse strains with contrasting susceptibility to obesity, we subjected the OP B6 and the OR 129 to either a CD or a HFD protocol for 16 wk (Figure 1 A). B6 mice increased their BW from 20 g to 38 g after HFD treatment compared to CD (Figure 1 B; p < 0.001), while no differences were noted in 129 mice with an average BW of 24 g in both groups (Figure 1 B). Moreover, a significant increase in FM was noted in B6 mice treated with HFD (HFD-fed B6, Figure 1C; p < 0.0001), as well as for AI (Figure 1 D; p < 0.0001). A slight increase in LM (Figure 1 E; p < 0.01) was also noted in B6 obese mice compared to their lean counterparts. Importantly, 129 mice fed a HFD (HFD-fed 129) presented significantly higher FI, with an average value of 104.8 kcal, compared to HFD-fed B6, which consumed an average of 62.1 kcal (Figure 1 F; p < 0.0001). No changes were noted between the CD groups in both strains (data not shown). Subsequently, in the GTT, we analysed insulin activity after glucose administration and monitored the time required for glucose levels to return to the baseline levels. In the ITT, plasma glucose levels were monitored after insulin injection [49]. In both tests glucose levels were recorded before the first injection and at 10, 20, 40, 60, 80, 100, and 120 min post-injection. In HFD-fed B6, glucose plasma levels remained significantly higher compared to the CD in both GTT and ITT (Figure 1 G, H). Next, after plotting glucose levels throughout time, we observed increased area under the curve (AUC) in HFD-fed B6, for both GTT and ITT (Figure 1 G-H; p < 0.01; p < 0.05, respectively). These results indicate a slower rate of glucose utilisation and insulin intolerance in OP mice (Figure 1 G, H). In contrast, 129 mice subjected to dietary protocols presented similar glucose values after challenging them with glucose or insulin and unchanged AUC between dietary groups, indicating normal glucose metabolism (Figure 1 G, H). Finally, leptin plasma levels increased in both strains treated with HFD, compared to CD, with greater changes noted in B6 mice (Figure 1 I; p < 0.0001 for B6 and p < 0.01 for 129). Thus, despite lower caloric intake, B6 mice challenged with HFD presented increased BW, leptin levels and glucose intolerance compared to the CD group, whereas 129 mice did not present any significant changes when challenged with different diets.

### 3.2 Obesity prone mice exhibit increased fat expansion and activation of inflammatory responses in gonadal fat during diet induced obesity

**Figure 2:**
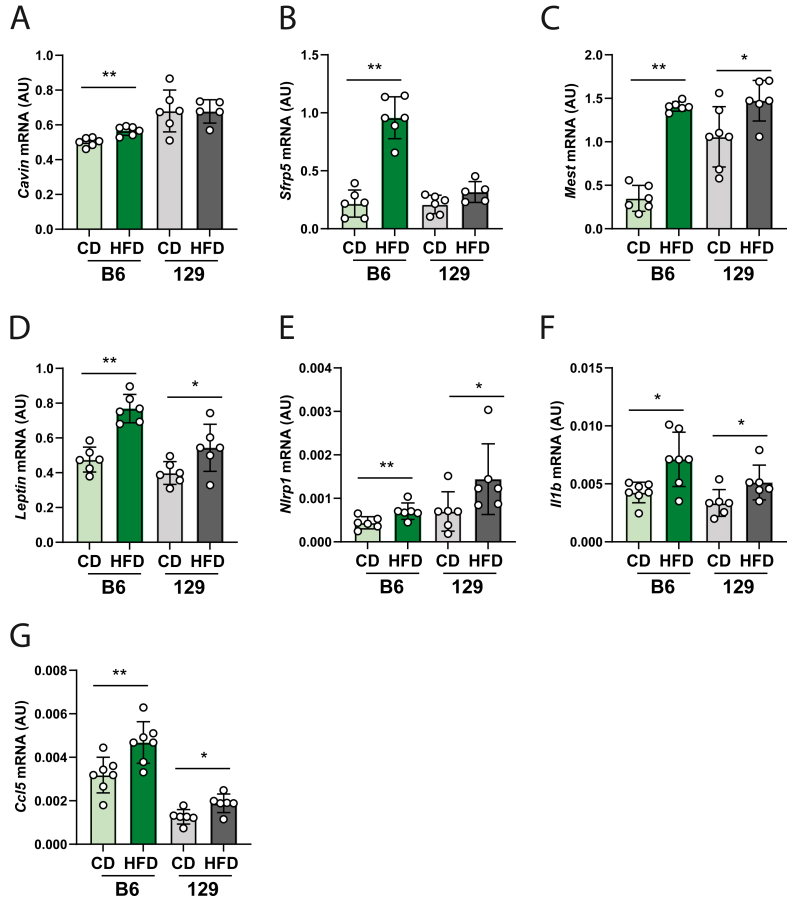
**Diet induced obesity promotes the expression of white adipose tissue expansion genes and proinflammatory markers in gonadal fat of obesity prone B6 mice**. Levels of mRNA transcription of gonadal fat (GON) expansion genes: (A) *Polymerase I and transcript release factor* (*Cavin*), (B) *Secreted frizzled-related protein 5* (*Sfrp5*), (C) *Mesoderm specific transcript* (*Mest*), (D) *Leptin*; and the proinflammatory cytokines: (E) *NLR Family Pyrin Domain Containing 1* (*Nlrp1*), (F) *Interleukin 1 beta* (*Il1b*), (G) *C–C motif chemokine ligand 5* (*Ccl5*) in obesity prone C57BL/6J (B6) and obesity resistant 129S1/SvlmJ (129) mice fed either chow diet (CD) or high fat diet (HFD) for 16 weeks. Fat expansion markers and leptin levels were measured by quantitative real-time PCR (RT-PCR) with TaqMan probes and primers using a standard curve generated from pooled RNA isolated from white adipose tissue and normalised to *cyclophilin b* expression level. Expression of proinflammatory markers was measured using SYBR Green real-time PCR and normalised to the averaged expression of *cyclophilin b* and *beta-2-microglobulin* (*b2m*). In each assay, both target and housekeeping gene were run simultaneously and in duplicate. AU- arbitrary units (results presented as mean ± standard deviation). The differences between groups were analysed using Mann–Whitney test. N = 6–8. Light green bars- B6 CD group, green bars- B6 HFD group, grey bars- 129 CD group, dark grey bars- 129 HFD group. Asterisks indicate significant differences: ∗p < 0.05; ∗∗p < 0.01. (For interpretation of the references to color in this figure legend, the reader is referred to the Web version of this article.)

Next, we profiled the expression of metabolic and proinflammatory markers in GON from B6 and 129 strains after DIO. Regarding fat expansion, we found that *Polymerase I and transcript release factor* (*Cavin*), as well as *Secreted frizzled-related protein 5* (*Sfrp5*), were significantly increased in GON from HFD B6 mice compared to their lean counterparts (Figure 2 A; B; p < 0.01 for both), while no changes were noted in 129 mice. Furthermore, the expression of *Mesoderm specific transcript* (*Mest*) was increased in both strains after HFD compared to CD, with more prominent changes noted in GON from HFD-fed B6 (Figure 2C; p < 0.01 for B6 and p < 0.05 for 129). The same was observed for *leptin* mRNA, which was upregulated in HFD-fed B6 (Figure 2 D; p < 0.01 for B6 and p < 0.05 for 129). A decrease in *S-phase kinase associated protein 2* (*Skp2*) mRNA expression was noted in HFD-fed B6 mice (Figure 2 Suppl 1 A; p < 0.01), with no changes detected in 129 mice (Figure 2 Suppl 1 A). Subsequently, the levels of proinflammatory cytokines were assessed. The mRNA levels of *NLR Family Pyrin Domain Containing 1* (*Nlrp1), Interleukin 1 beta* (*Il1b)* and *C–C motif chemokine ligand 5* (*Ccl5*) were significantly increased in both B6 (Figure 2 E-G; p < 0.01, p < 0.05, p < 0.01 accordingly) and 129 fed a HFD (Figure 2 E-G; p < 0.05). Moreover, the expression of *Ccl2* was increased exclusively in GON from HFD-fed B6 mice (Figure 2 Suppl 1 B; p < 0.001), whereas *Tumour necrosis factor alpha* (*Tnfa)* was increased in HFD-fed 129 (Figure 2 Suppl 1C; p < 0.01) and with no changes in B6 mice. Next, we analysed the expression of markers linked to steroidogenesis. No changes were detected in the mRNA transcription of *Star*, *Hsd3b*, or *Cyp17a1* (Figure 2 Suppl 1 D-F). The mRNA level of *Hsd17b* was decreased in GON isolated from HFD-fed B6 (Figure 2 Suppl G, p < 0.01), with no changes detected in 129 mice. Thus, in OP B6 females, DIO leads to increased transcription of genes linked to fat expansion and mounting inflammatory response.

### 3.3 Dysregulation of ovarian steroidogenesis in obesity prone B6 mice is associated with decreased levels of lipid droplets and transcription of steroidogenic enzymes in the theca compartment

**Figure 3:**
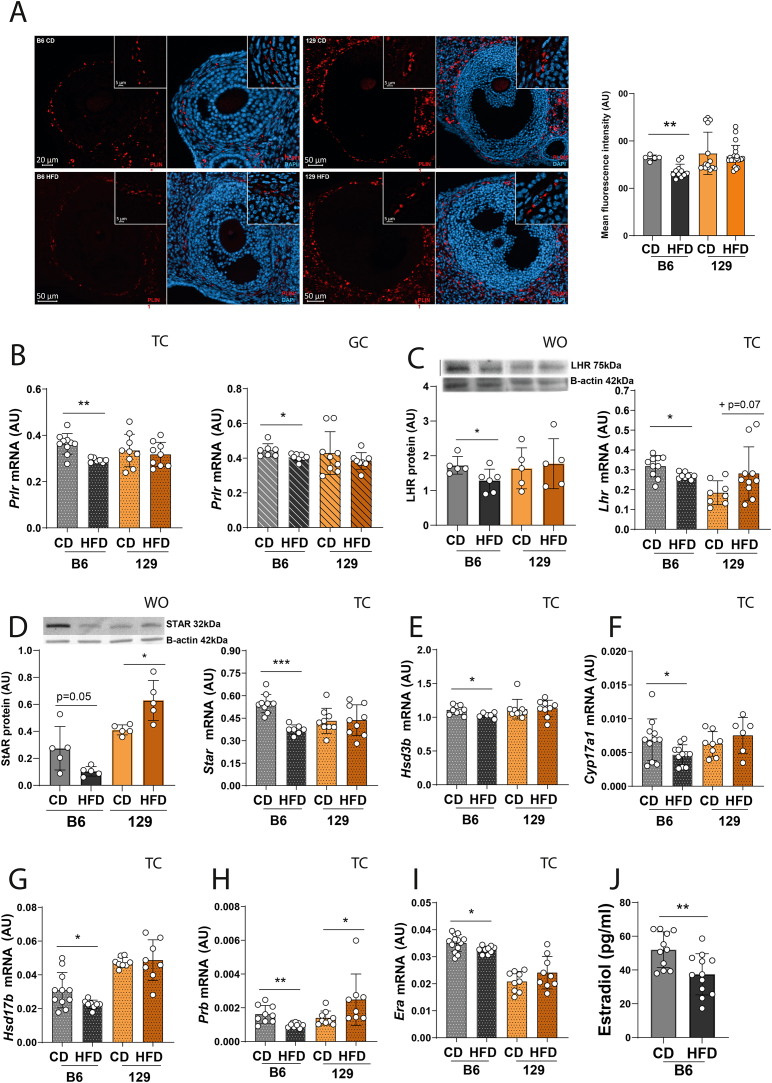
**The expression of gonadotropin receptors and steroidogenic enzymes is compromised in the theca compartment of ovaries collected from obese B6 mice**. Whole ovaries (WO), theca-enriched population (TC), the granulosa cells (GC) were collected from obesity prone (C57BL/6J, B6) and obesity resistant (129S1/SvlmJ, 129) mice, fed either chow diet (CD) or high fat diet (HFD) for 16 weeks following superovulation. (A) Immunofluorescent staining of perilipin 1 (PLIN 1) was performed in paraffin embedded ovaries of mice subjected to the dietary protocol. PLIN 1 visualised in red was localised in the TC in ovarian follicles; nuclear counterstaining with DAPI in blue. The scale bar represents 5, 20 or 50 μm. Images shown are representative of 3 biological replicates, illustrating follicles from B6 CD (top left), B6 HFD (bottom left) 129 CD (top right) and 129 HFD (bottom right). The insert on the top right represents a magnified area of the main image. The bar plot on the right represents the mean fluorescence intensity in late antral follicles. Briefly, using Zeiss ZEN 3.7 software, both the theca layer and the mural granulosa cell layer were outline. Subsequently the background intensity was set for the GC layer as the minimum threshold signal, and the intensity in TC measured. Results represent the average intensity across antral follicles in three ovarian sections obtained from different mice in each treatment. Abundance of (B) *Prolactin receptor* (*Prlr*) mRNA in TC fraction (left graph) and GC compartment (right graph), (C) Luteinizing hormone receptor (LHR) protein expression in WO (left graph) and mRNA level in TC (right graph). Expression of steroidogenic markers and transcription factors in WO and TC, (D) Steroidogenic acute regulatory protein (StAR) protein in WO and mRNA levels in TC, and mRNA assessment of (E) *Hydroxysteroid dehydrogenase 3 beta* (*Hsd3b*), (F) *Cytochrome P450 family 17 subfamily A member 1* (*Cyp17a1*), (G) *Hydroxysteroid dehydrogenase 17 beta* (*Hsd17b*), (H) *Progesterone receptor isoform b* (*Prb*), (I) *Estradiol receptor alpha* (*Era*) in TC. (J) Estradiol plasma levels measured by enzyme linked immunosorbent assay (ELISA) in B6 mice submitted to CD and HFD for 16 weeks. Levels of mRNA measured with SYBR Green real-time PCR and normalised by the housekeeping genes *Ribosomal protein L32* (*Rpl32*, WO) or *Eukaryotic translation initiation factor 5A* (*Eif5a*, TC and GC). In each assay, both target and housekeeping genes were run simultaneously and in duplicate. Protein expression was measured by western blot (WB) and normalised by B-actin. AU- arbitrary units (results presented as the mean ± standard deviation). The differences between groups were analysed using Mann–Whitney test. N = 6–8 for real-time PCR and N = 4 for WB. Grey bars- B6 CD group, black bars- B6 HFD group, orange bars- 129 CD group, brown bars- 129 HFD group. Plain bars- WO, dotted bars- TC, stripped bars- GC. Asterisks indicate significant differences: ∗p < 0.05; ∗∗p < 0.01; ∗∗∗p < 0.001. (For interpretation of the references to color in this figure legend, the reader is referred to the Web version of this article.)

In the following experiment, we characterised the ovarian performance of OP and OR mice after DIO. After SO (Figure 3 Suppl 1 A) and ovary collection, histological analysis revealed the presence of follicles at all developmental stages across strains and treatments (Figure 3 Suppl 1 B). Following ovarian puncturing, we counted a lower number of GVs isolated from HFD treated mice, with average of 42 GV oocytes in B6 mice fed a CD (CD-fed B6) compared to 28 oocytes in HFD-fed B6, and 59 oocytes in 129 mice fed a CD (CD-fed 129) compared to 31 oocytes in the HFD group (Figure 3 Suppl 1C; p < 0.0001 for both, Supplementary Table 3). After performing SO, no changes were observed in the number of MII oocytes retrieved (Figure 3 Suppl 1C, Supplementary Table 3), the number of 2-cell embryos generated after IVF (Figure 3 Suppl 1 D, Supplementary Table 3), or their progression to the blastocyst stage (Figure 3 Suppl 1 E, Supplementary Table 3). Overall, despite decreased GV count, MII oocyte retrieval and embryo development were preserved in both OP and OR mice after DIO.

A major focus of our study was to characterise the effects of DIO on ovarian steroidogenesis in OP and OR mice. We started by identifying PLIN1, a marker of lipid droplets that ensures steroid precursors availability [18,54], distribution in the ovaries of OP and OR by IF (Figure 3 A). Distinct staining was initially observed in the theca compartment of antral follicles in all studied groups (Figure 3 Suppl 2 A- D). After quantifying the signal and confirming the specificity of the signal (Figure 3 Suppl 2 E, F), we noted lower mean fluorescence intensity in the TC from HFD-fed B6 compared to CD (Figure 3 A; p < 0.01), but no changes in 129 mice (Figure 3 A). Next, we used WO, TC and GC fractions isolated from the ovaries of OP and OR mice for further analysis of steroidogenesis regulation (Figure 3 Suppl 3 A). The purity of the ovarian fractions was confirmed by observing increased mRNA levels of *Cyp17a1* and *GLI family zinc finger 3* (*Gli3*; TC markers), and decreased levels of *Cyp19a1* and *Follicle stimulating hormone receptor* (*Fshr,* GC markers) in the TC fraction (Figure 3 Suppl 3 B; p < 0.0001), and decreased levels of *Cyp17a1* and *Gli3*, but increased levels of *Cyp17a1* and *Fshr* in the GC fraction (Figure 3 Suppl 3C; p < 0.0001). We further profiled the activity of steroidogenesis modulators (Figure 3 Suppl 3 D). The mRNA of *prolactin receptor* (*Prlr*) was decreased in WO, TC and GC of HFD-fed B6 (Figure 3 Suppl 3 E, Figure 3 B; p = 0.06, p < 0.01, p < 0.05 respectively), contrasting with its upregulation exclusively in WO from HFD-fed 129 (Figure 3 Suppl 3 E; p < 0.01). Protein analysis showed decreased expression of LHR in WO of HFD-fed B6, as well as *Lhr* mRNA levels in TC from the same group (Figure 3C; p < 0.05). Importantly, we also observed a consistent downregulation of mRNA levels of *Star*, *Hsd3b, Cyp17a1* and *Hsd17b* in TC from HFD-fed B6 (Figure 3 D- G; p < 0.05). Furthermore, *Star* mRNA and StAR protein expression were downregulated in WO from HFD-fed B6 (Figure 3 Suppl 3 F, Figure 3 D; p = 0.05), but no changes were found for the aforementioned genes in WO, TC or GC from HFD-fed 129 (Figure 3D–G). We next characterised the behaviour of steroid hormone receptors and confirmed that the dysregulation in steroidogenesis in TC from HFD treated B6 mice was associated with decreased mRNA transcription of *progesterone receptor isoform b* (*Prb*) and *estradiol receptor isoform a (Era)* (Fig. 3H, I; p < 0.01, p < 0.05). Furthermore, *Er isoform b* (*Erb*) was also decreased in GC from HFD-fed B6 (Figure 3 Suppl 3 G; p < 0.05). Conversely, *Prb* was increased in TC from HFD-fed 129 (Figure 3H; p < 0.05), despite the contrasting profile in WO (Figure 3 Suppl 3H; p < 0.05) and no changes in GC from 129 HFD (Figure 3 Suppl 3H). No changes were observed in *Era* (Figure 3 I) and *Erb* in 129 mice (Figure 3 Suppl 3 G). Finally, the mRNA levels of the steroidogenesis-associated transcription factor *Nr5a1* were decreased in TC from HFD-fed B6 (Figure 3 I, p < 0.05), whereas *Gata4* mRNA was upregulated in HFD-fed 129 (Figure 3 J, p < 0.05). In line with the previous observations, we also found the levels of E2 to be decreased in plasma from HFD-fed B6 mice (Figure 3 J; p < 0.01). Overall, decreased circulating levels of E2 in HFD-fed B6 mice are due to compromised ovarian response to gonadotropins, and decreased steroidogenic activity particularly in the TC compartment.

### 3.4 Nodal signalling is suppressed in the theca compartment of obesity prone mice

**Figure 4:**
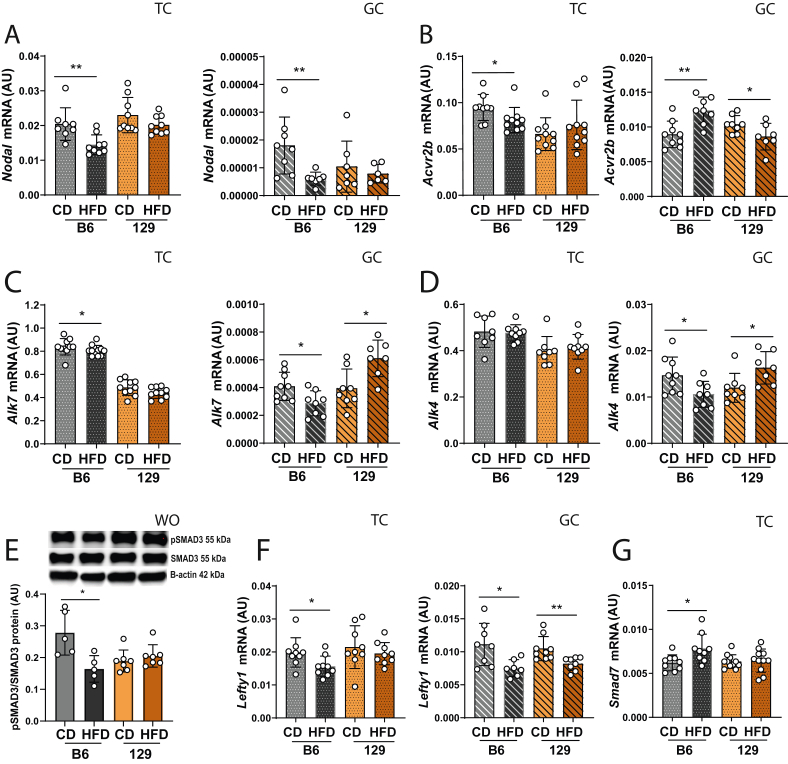
**Diet-induced obesity downregulates Nodal signalling pathway in the theca compartment of ovaries from obesity-prone B6 mice**. Whole ovaries (WO), theca (TC) and granulosa cells (GC) were collected from ovaries of C57BL/6J (B6) and 129S1/SvlmJ (129) mice fed either chow diet (CD) or high-fat diet (HFD) for 16 weeks. The mRNA transcription was analysed by real time PCR for: (A) *Nodal*, (B) *Activin a receptor type 2b* (*Acvr2b*), (C) *Activin a receptor type 1c* (*Alk7*), (D) *Alk4*. (E) Phosphorylated SMAD3 (pSMAD3) levels in WO were analysed by western blot (WB) and normalised to total SMAD3. mRNA levels of (F) *Left-right determination factor 1* (*Lefty 1*) and (F) *Smad7* were also quantified in TC and GC. Levels of mRNA measured with SYBR Green real-time PCR and normalised by the housekeeping gene *Eukaryotic translation initiation factor 5A* (*Eif5a*). In each assay, both target and housekeeping genes were run simultaneously and in duplicate. Protein expression assessed by western blot and normalised by B-actin. AU- arbitrary units (results presented as the mean ± standard deviation). The differences between groups were analysed using Mann–Whitney test. N = 6–8 for real-time PCR and N = 4 for WB. Grey bars- B6 CD group, black bars- B6 HFD group, orange bars- 129 CD group, brown bars- 129 HFD group. Plain bars- WO, dotted bars- TC, stripped bars- GC. Asterisks indicate significant differences: ∗p < 0.05; ∗∗p < 0.01. (For interpretation of the references to color in this figure legend, the reader is referred to the Web version of this article.)

We next profiled the expression of Nodal signalling components in the ovaries of OP and OR mice after DIO, in light with the putative association between the pathway and compromised steroidogenesis in obese mice. The analysis of the ovarian fractions showed the downregulation of *Nodal* and its receptors, *Activin receptor type 2 B* (*Acvr2b*)*,* and *Alk7* in the TC from HFD-fed B6 (Figure 4 A-C; p < 0.01, p < 0.05, p < 0.05 accordingly). No changes were observed in *Alk4* mRNA isolated from TC in either strain (Figure 4 D). Regarding the GCs, *Nodal*, *Alk7* and *Alk4* mRNA levels were downregulated in HFD-fed B6 (Figure 4 A, C, D; p < 0.01; p < 0.05 accordingly), whereas *Alk7* and *Alk4* were upregulated in HFD-fed 129 (Figure 4C, D; p < 0.05). Finally, *Acvr2b* mRNA was upregulated in GC from HFD-fed B6 but downregulated in HFD-fed 129 (Figure 4 B; p < 0.01, p < 0.05 accordingly). Nodal signalling modulation was confirmed by assessing the phosphorylation levels of SMAD3 (pSMAD3) in OVA, which showed a significant downregulation in HFD-fed B6 mice (Figure 4 E, p < 0.05). Notably the expression of *Left right determination factor 1* (*Lefty1*), an antagonist of the Nodal pathway [55], was decreased in both TC and GC of HFD-fed B6 (Figure 4 F, p < 0.05 both) and in GC of HFD-fed 129 (Figure 4 F; p < 0.01). Finally, we analysed the expression of the Nodal signalling regulator, *Smad7* [56], confirming its mRNA upregulation exclusively in the TC of HFD-fed B6 (Figure 4 G, p < 0.05). In conclusion, our results revealed the downregulation of Nodal signalling pathway components in the TC of ovaries collected from OP mice following DIO.

### 3.5 Leptin hyperactivation inhibits nodal signalling in the ovaries of obese mice

**Figure 5:**
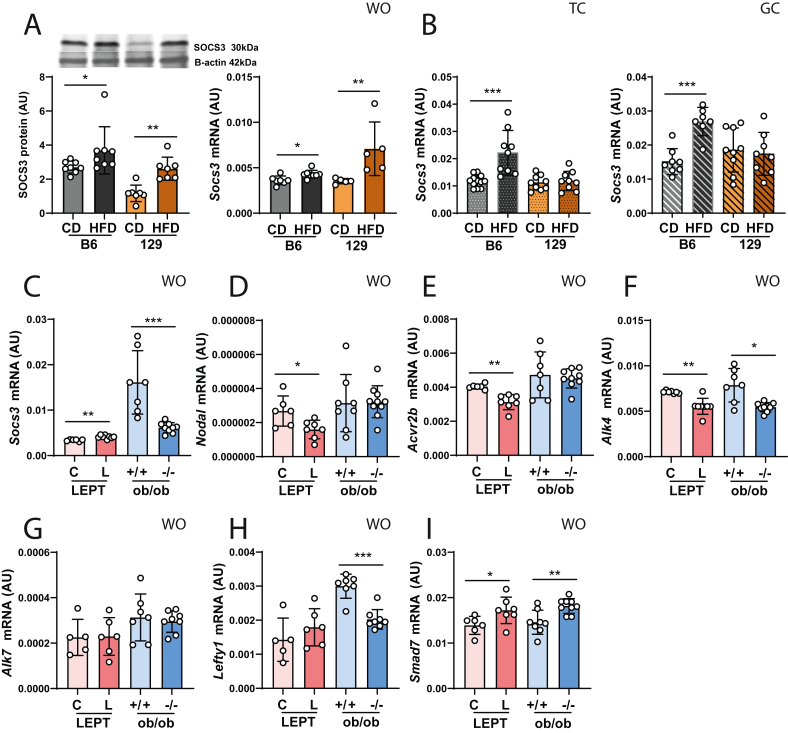
**SOCS3 activation in the ovaries of obese mice inhibits Nodal signalling pathway.** Obesity prone (C57BL/6J, B6) and obesity resistant (129S1/SvlmJ, 129) mice were fed either chow diet (CD) or high fat diet (HFD) for 16 weeks, followed by the collection of the whole ovaries (WO), the theca-enriched (TC) compartment and granulosa cell (GC) compartment. WO were collected from 12 week old mice pharmacologically treated with leptin (LEPT) or saline (C) for 16 days; and 12 week old mice with genetic leptin deficiency (ob/ob) and their wild type counterparts. Abundance of (A) Suppressor of cytokine signalling 3 (SOCS3) protein (left graph) and mRNA (right graph) in WO; (B) *Socs3* mRNA in the TC (left graph) and GC (right graph) collected from B6 and 129. mRNA transcription of: (C) *Socs3*, (D) *Nodal*, (E) *Activin a receptor type 2b* (*Acvr2b*), (F) *Activin a receptor type 1b* (*Alk4*), (G) *Activin a receptor type 1c* (*Alk7*), (H) *Left-right determination factor 1* (*Lefty 1*) and (I) *SMAD family member 7* (*Smad 7*) in WO of mice collected LEPT and ob/ob protocols. Levels of mRNA measured with SYBR Green real-time PCR and normalised by the housekeeping genes *Ribosomal protein L32* (*Rpl32*) (WO) and *Eukaryotic translation initiation factor 5A* (*Eif5a*) (TC and GC). In each assay, both target and housekeeping genes were run simultaneously and in duplicate. Protein expression assessed by western blot and normalised by B-actin. AU- arbitrary units (results presented as the mean ± standard deviation). The differences between groups were analysed using Mann–Whitney test. N = 6–8 for real-time PCR and N = 4 for WB. Grey bars- B6 CD group, black bars- B6 HFD group, orange bars- 129 CD group, brown bars- 129 HFD group. Plain bars- WO, dotted bars- TC, stripped bars- GC. Light pink bars- LEPT C group, pink bars- LEPT L group, light blue bars-ob/ob +/+ group, blue bars-ob/ob −/− group. Asterisks indicate significant differences: ∗p < 0.05; ∗∗p < 0.01; ∗∗∗p < 0.001. (For interpretation of the references to color in this figure legend, the reader is referred to the Web version of this article.)

We next asked whether the crosstalk between leptin and Nodal signalling pathways in the ovaries of obese mice could lead to compromised steroidogenesis (Figure 5 Suppl 1 A). Our previous results demonstrated the establishment of leptin resistance in the ovaries of HFD-fed B6 mice, through the upregulation of SOCS3 expression [3]. Expectedly, we confirmed the upregulation of SOCS3 protein and mRNA levels in WO of HFD-fed B6 (Figure 5 A; p < 0.05) and HFD-fed 129 (Figure 5 A; p < 0.01). Furthermore, *Socs3* mRNA was upregulated in TC and GC in HFD-fed B6 (Figure 5 B; p < 0.001), but not in HFD-fed 129 (Figure 5 B). SOCS3 is known to inhibit the expression of *Il6* [57], which mRNA was significantly decreased in WO and TC from HFD-fed B6 mice (Figure 5 Suppl 1 B; p < 0.05) or in TC from HFD-fed 129 (Figure 5 Suppl 1 B; p < 0.05). In order to confirm the crosstalk between SOCS3 and Nodal in the ovaries of DIO mice, we used a lean mouse model with pharmacological hyperleptinemia (LEPT) [3] and the extremely obese mouse model lacking leptin (*ob/ob*) [58]. The WO of LEPT mice presented a consistent upregulation of SOCS3 protein (Figure 5 Suppl 1C; p < 0.01) and mRNA (Figure 5C; p < 0.01), whereas the *ob/ob* −/− showed decreased *Socs3* mRNA levels (Figure 5C; p < 0.001). Furthermore, *Socs3* was upregulated in the TC of LEPT mice (Figure 5 Suppl 1C; p < 0.05). Finally, we observed decreased mRNA levels of *Nodal*, *Acvr2b* and *Alk4* in WO from LEPT mice (Figure 5 D- F; p < 0.05, p < 0.01, p < 0.01), but no changes in *ob/ob* −/− mice except for the downregulation of *Alk4* (Figure 5 F; p < 0.05). No changes were observed in *Alk7* in both models (Figure 5 G). Despite no changes in *Lefty1* mRNA in WO of LEPT mice, *Smad7* was found to be upregulated (Figure 5H–I). In contrast, *Lefty1* mRNA levels were decreased in WO of *ob/ob* −/− mice (Figure 5H; p < 0.001), whereas *Smad7* mRNA was increased (Figure 5 I; p < 0.01). These results highlight the intricate link between leptin signalling hyperactivation and decreased Nodal activity in the ovaries of obese mice.

### 3.6 In vitro treatment of ovarian explants with nodal restores the transcriptional profile of steroidogenic enzymes in the TC compartment of obesity prone mice

**Figure 6:**
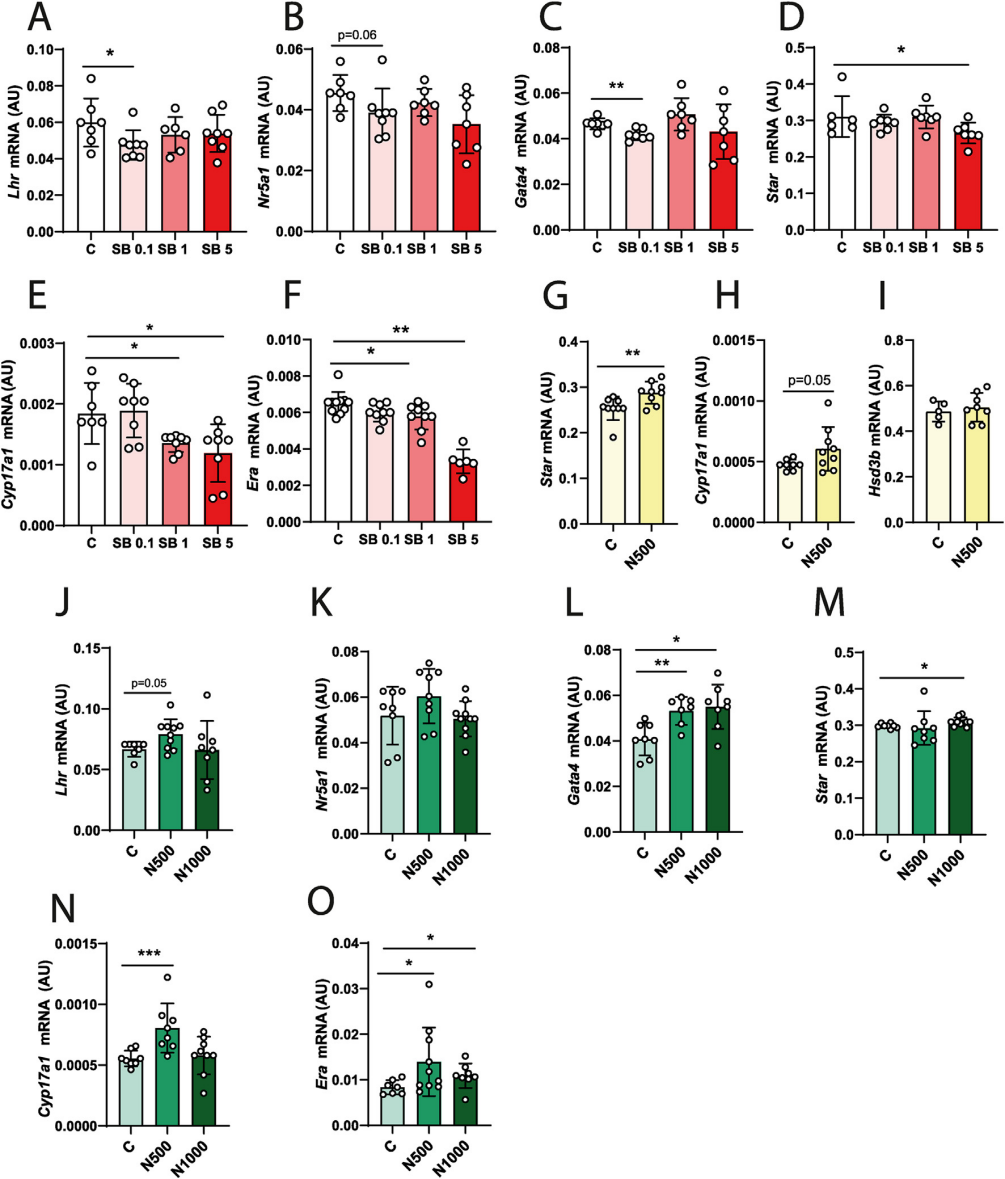
**Nodal treatment *in vitro* rescues the downregulation of steroidogenic genes in the TC compartment of obese mice**. Obesity prone (C57BL/6J, B6) mice were fed either chow diet (CD, sections A-I) or high fat diet (HFD, sections J-O) for 16 weeks, followed by the collection of whole ovaries (WO) and ovarian explants cultured for 12 h with: Nodal signalling inhibitor SB431542 (SB) at 0.1, 1 or 5 μM,; or 500, Nodal at 1000 ng/ml. Control group represented by C. After culture, ovarian explants were punctured and the theca-enriched (TC) was collected for mRNA analysis of: (A, J) *Luteinizing hormone receptor (Lhr),* (B, K) *Nuclear receptor subfamily 5 group A member 1* (*Nr5a1*), (C, L) *Gata binding protein 4* (*Gata4*), (D, G, M) *Steroidogenic acute regulatory protein* (*Star*), (E, H, N) *cytochrome P450 family 17 subfamily A member 1* (*Cyp17a1*), (F, O) *Estradiol receptor alpha* (*Era*) and *Hydroxy-steroid dehydrogenase 3b* (*Hsd3b*). Levels of mRNA normalised to *Eukaryotic translation initiation factor 5A* (*Eif5a*) expression run simultaneously with the gene of interest. AU- arbitrary units (results presented as the mean ± standard deviation). The differences between groups were analysed using Mann–Whitney test. N = 6–8. White bars- B6 CD group, grading red bars- B6 CD group treated with SB431542, light green bars- B6 HFD group, grading green bars- B6 HFD group treated with Nodal. Asterisks indicate significant differences: ∗p < 0.05; ∗∗p < 0.01; ∗∗∗p < 0.001. (For interpretation of the references to color in this figure legend, the reader is referred to the Web version of this article.)

To confirm the supportive role of Nodal signalling on TC steroidogenic activity (Figure 6 Suppl 1 A), we firstly treated ovarian explants collected from CD-fed B6 with SB (0.1, 5 and 100 μM), a selective inhibitor of Alk4, Alk5, Alk7 receptor activity [59] or Nodal 50, 100 and 500 ng/ml (Figure 6 Suppl 1 B). After measuring cell viability, we confirmed that the SB treatment doses below 5 μM did not compromise tissue viability (Figure 6 Suppl 1C). Furthermore, the increased expression of TC-specific genes confirmed the purity level of our TC-enriched fractions collected during the *in vitro* experiments (Figure 6 Suppl 1 D). The ability of SB to modulate *Alk5* reders TGFb1 pathway sensitive to the pharmacological actions of SB. After confirming the reduction in pSMAD3 levels in OVA following SB treatment (Figure 6 Suppl 1 E, p < 0.05), the specific inhibitory effects of SB on Nodal signalling were demonstrated by the analysis of Nodal-specific genes downstream of pSMAD3 activation in TC. Importantly, the mRNA of Nodal-specific co-receptor *Cripto, EGF-CFC Family Member (Cripto)* [60] was downregulated by SB 5 μM (Figure 6 Suppl 1 F, p < 0.01), whereas Nodal signalling antagonist *Left 1* [55] was upregulated by SB treatment (Figure 6. Suppl 1 G, p < 0.05). In contrast, the transcription of TGFb1-specific genes like *Tgfb1, Alk5* or *Fibronectin 1 (Fn1)* remained unchanged after SB treatment (Figure 6. Suppl 1 H-J), further corroborating the Nodal specific actions of SB in our system. Concerning steroidogenesis regulation, the treatment of ovarian explants collected from CD-fed B6 with SB revealed a dose-dependent response to the treatment, with SB 0.1 μM downregulating the mRNA of *Lhr* (Figure 6 A; p < 0.05), *Nr5a1* (Figure 6 B, p = 0.06) and *Gata4* (Figure 6C; p < 0.01), while SB 1 μM reduced the transcription of *Cyp17a1* (Figure 6 E; p < 0.05) and *Era* (Figure 6 F; p < 0.05) in TC. Furthermore, SB 5 μM significantly downregulated the mRNA levels of *Star* (Figure 6 D; p < 0.05), *Cyp17a1* (Figure 6 E; p < 0.05) and *Era* (Figure 6 F; p < 0.01). To confirm the supportive effects of Nodal on steroidogenesis in physiological conditions, we also treated ovarian explants collected from mice fed a CD with 500 ng/ml of Nodal (Figure 6 Suppl 1 B). The transcription of both *Star* and *Cyp17a1* mRNA was increased (Figure 6 G, p < 0.001; Figure 6H, p = 0.06), with no changes in *Hsd3b* mRNA (Figure 6 I). Finally, we interrogated whether the treatment of HFD-fed B6 ovaries with Nodal would restore steroidogenesis, particularly at the TC level (Figure 6 suppl 1 K). Following cell viability assessment (Figure 6 Suppl 1 L), we found that the *in vitro* supplementation of HFD-fed B6 ovaries with Nodal 500 ng/ml upregulated the mRNA levels of *Lhr* (Figure 6 J; p = 0.05); *Cyp17a1* (Figure 6 N; p < 0.001), *Era* (Figure 6 O; p < 0.05), and *Gata4* (Figure 6 L; p < 0.01) in TC. Furthermore, treatment with 1000 ng/ml of Nodal upregulated the transcription of *Star* (Figure 6 M; p < 0.05) and *Era* (Figure 6 O; p < 0.05) in TC. No changes were seen in *Nr5a1* transcription (Figure 6 K). This experiment highlights the strong association between inhibited Nodal activity and compromised steroidogenesis in the TC compartment of ovaries from obese mice.

## 4 Discussion

The present study sheds light on the underlying mechanisms compromising ovarian steroidogenesis in obese mothers. Using two mouse models of maternal obesity with contrasting susceptibility to DIO, we found that the OP mice presented hyperleptinemia and decreased circulating levels of E2, associated with reduced ovarian protein expression of the steroidogenic enzyme StAR. Furthermore, the transcription of *Star*, *Hsd3b*, *Cyp17a1*, and *Hsd17b* were drastically reduced in the TC compartment of ovaries collected from HFD-fed B6 mice. Mechanistic studies performed *in vitro* with ovarian explants revealed that decreased steroidogenesis in HFD-fed B6 mice resulted from compromised Nodal signalling activity in TC, which was inhibited by leptin-mediated upregulation of *Smad7*. Additionally, Nodal treatment of ovarian explants from HFD-fed B6 mice led to increased transcription of *Star* and *Cyp17a1* in TC. Overall, we confirmed that greater susceptibility to obesity in mothers leads to impaired ovarian steroidogenesis through decreased Nodal signalling activity and reduced support of steroidogenic machinery in the TC compartment, primarily driven by leptin-mediated upregulation of *Smad7*.

Previous research has revealed the molecular mechanisms determining BW gain and adipose tissue expansion in male mice under obesogenic conditions [4]. Presently, female B6 mice treated with HFD presented significant gains in BW and increased transcription of *Cavin* and *Sfrp5* in GON, two established adipose tissue expansion genes [61]. Additionally, the same tissue presented increased transcription of the proinflammatory factors *Nlrp1* and *Il1b* [62], as well as the chemokine *Ccl5*, known to mediate macrophage recruitment [63]. Glucose metabolism was significantly altered in HFD-fed B6 mice, characterised by hyperglycaemia after glucose challenge and decreased insulin sensitivity. This could potentially contribute to endocrine imbalances and the dysregulation of ovarian function in obese mothers [64]. Conversely, HFD-fed 129 presented no changes in BW and FM, or alterations in glucose metabolism, despite the upregulation of proinflammatory genes in GON. Overall, B6 OP female mice presented an exuberant response to DIO, reflecting their increased susceptibility to obesity, in contrast to 129 OR female mice.

We next investigated whether the phenotypic discrepancies between OP and OR mice could lead to distinct reproductive outcomes. Ovarian histology revealed no changes between OP and OR strains following DIO. Nonetheless, a decreased number of GV oocytes was retrieved from both OP and OR mice fed a HFD after ovarian mechanical disruption, evidencing compromised ovarian function. Conversely, after SO, no differences were observed in the number of MII oocytes collected, or in the number of 2-cell embryos and blastocysts generated in OP and OR mice fed with HFD. Our results indicate that HFD treatment in both OP and OR mice may dysregulate folliculogenesis and compromise the oocyte pool, despite no major effects observed on the regulation of follicular recruitment, ovulation and early embryo development.

Ovarian steroidogenesis leads to the production of hormones like progestins, androgens and estrogens, responsible for controlling folliculogenesis and oocyte development [65]. The primary ovarian steroidogenic unit is the TC compartment, in which lipid droplets containing cholesterol (the main precursor of steroidogenesis) are accumulated [18]. Progesterone and testosterone are synthesised in the TC and posteriorly transferred to the GC for further conversion into E2 [20]. Numerous reports have demonstrated the detrimental effects of maternal obesity on ovarian steroidogenesis [66,67]. We presently found decreased transcription of *Prlr* in the TC of ovaries collected from HFD-fed B6 mice. Prolactin is known to regulate progesterone synthesis [68]. Importantly, disrupted prolactin signalling was shown to reduce the expression of another major orchestrator of steroidogenesis in mouse ovaries, the LHR [69]. Moreover, *in vitro* treatment of human TC with LH increased the transcription of *Cyp11a1*, *Hsd3b*, and *Cyp17a1* [70]. In our study, the mRNA levels of *Prlr* and *Lhr* were decreased in the TC of HFD-fed B6, demonstrating compromised modulation of steroidogenesis. Furthermore, we also observed decreased levels of PLIN1 in TC of the same group. Within the TC, PLIN1 coats lipid droplets [18]. Therefore, the decreased staining of PLIN1 observed in the TC of antral follicles from HFD-fed B6 suggest reduced amount of lipid precursors necessary for steroidogenesis initiation. Subsequently, we saw a dramatic downregulation in the transcription of *Star*, *Hsd3b*, *Cyp17a1* and *Hsd17b* in the TC, and decreased StAR protein expression in whole ovaries from HFD-fed B6 mice. Finally, the mRNA levels of StAR and 3bHSD-regulating transcription factors *Nr5a1* [22,23] and *Gata4* [23,24], were also decreased in the TC of the same group. In conclusion, our findings revealed that maternal obesity compromises ovarian steroidogenesis by reducing the amount of lipid droplets and diminishing the expression of steroidogenic enzymes, particularly in the TC compartment of HFD-fed B6 mice.

Few studies have previously reported the involvement of Nodal in ovarian steroidogenesis [44,71]. Presently, we observed that the *in vitro* treatment of ovaries from lean mice with Nodal upregulated *Star* and *Cyp17a1* mRNA transcription in the TC. Conversely, decreased ovarian steroidogenesis in HFD-fed B6 mice was associated with reduced transcriptional activity of Nodal signalling components in the TC compartment. Importantly, our results also showed that the *in vitro* treatment of ovarian explants from HFD-fed B6 with Nodal upregulated the mRNA level of *Star* and *Cyp17a1* in the TC. These findings revealed the molecular link between compromised ovarian steroidogenesis in obese mothers and decreased activity of the Nodal signalling in the TC. Furthermore, the pharmacological inhibition of Nodal signalling pathway, following the *in vitro* treatment of ovaries from lean mice with SB, downregulated *Gata4* transcription in the TC, whereas the treatment with Nodal elicited the opposite response, increasing *Gata4* mRNA levels. The link between GATA4 and Nodal was previously evidenced in embryonic human cardiomyocytes, in which increased transcription of *Gata4* was observed in response to endogenous Nodal [72]. Our findings imply that Nodal supports ovarian steroidogenesis through the expression of transcription factors like GATA4. To further validate the specific regulation of Nodal signalling by SB treatment, we observed a significant downregulation of the Nodal co-receptor *Cripto* in the TC compartment. Cripto is essential for facilitating Nodal interaction with its type I receptors, Alk4 and Alk7, as well as the type II receptor ACVRIIB [73]. Additionally, the upregulation of *Lefty1*, a known antagonist that competes with Nodal for its receptors, further corroborates the repression of Nodal signalling following SB treatment [55]. Conversely, the transcription of TGFb1-specific target genes *TGFb1*, *Alk5* [74], and *Fn1* [75] remained unchanged, confirming the selectivity of SB in targeting Nodal signalling without affecting TGFb1 pathway. In conclusion, decreased ovarian steroidogenesis in obese mothers may result from reduced Nodal activity in the TC and compromised expression of steroidogenic enzymes.

Several mechanisms were shown to regulate Nodal signalling, including Lefty1, an established antagonist of the pathway [55]. We found that the transcription of *Lefty1* was decreased in the TC from HFD-fed B6, suggesting the involvement of an alternative mechanism suppressing Nodal signalling in the ovaries of obese mothers. Our previous research highlighted the establishment of leptin resistance in the ovaries of DIO mice through the dramatic upregulation of SOCS3 expression. Presently, *Socs3* transcription was augmented in TC from HFD-fed B6. Furthermore, leptin signalling hyperactivation has previously been associated with the upregulation of SMAD7 expression [76], a protein known to mediate the crosstalk between STATs and SMADs signalling in various biological contexts, like early tumorigenesis [77,78]. We presently reported the upregulation of *Smad7* mRNA in TC from HFD-fed B6 mice, suggesting that leptin-mediated increase in *Smad7* expression could suppress Nodal signalling in the ovaries of obese mothers. Additionally, we saw increased transcription of *Smad7* in the ovaries of mice pharmacologically treated with leptin, followed by decreased transcription of Nodal signalling components *Nodal*, *Acvr2b* and *Alk4*. Thus, the hyperactivation of leptin in the TC compartment of ovaries from obese mothers may activate *Smad7* transcription, leading to the suppression of the Nodal signalling pathway and steroidogenesis.

**Figure 7:**
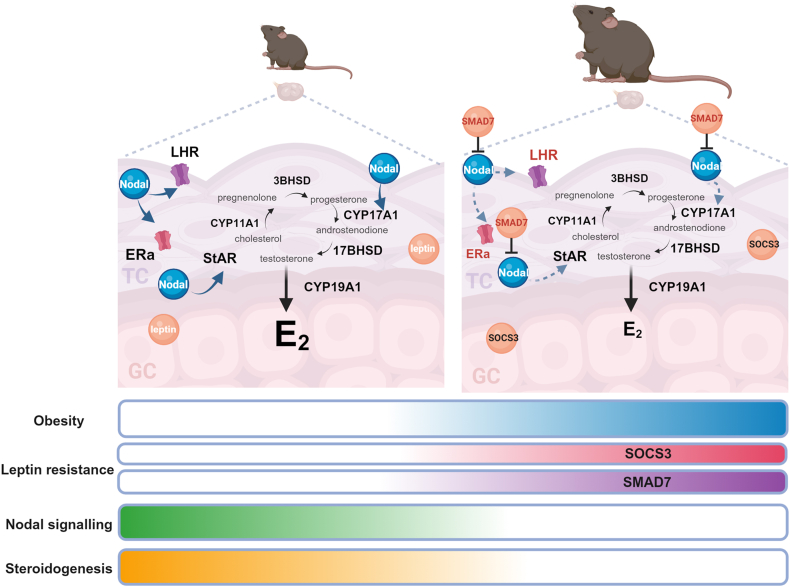
**Compromised steroidogenesis in the ovaries of obese mice is mediated by suppressed Nodal signalling in the TC compartment via SOCS3 and Smad7 increased activity**. Steroidogenesis is a complex process, primarily stimulated by luteinizing hormone (LH), that activates the cascade of signals within the theca cell (TC) compartment. Firstly, steroidogenic acute regulatory protein (StAR) facilitates cholesterol transport to the mitochondrial inner membrane. Then, mitochondrial enzymes convert intermediate products. Cholesterol is converted into pregnenolone by cytochrome P450 11A1 (CYP11A1), which is next converted into progesterone by 3B Hydroxysteroid dehydrogenase (3BHSD). CYP17A1 converts progesterone in to androstenedione, which is transformed into testosterone by 17BHSD. Finally, testosterone is transported from TC to the granulosa cells (GC), where the conversion into estradiol (E2) is done CYP19A1. E2 can bind to its receptor on TC- estradiol receptor isoform a (ERa). In mice fed chow diet (CD), normal body weight is maintained, and ovarian leptin and Nodal signalling are preserved, ensuring that steroidogenesis in the TC remains intact. Nodal supports the expression of luteinizing hormone receptor (LHR), ERa, StAR and CYP17A1. Contrarily, when females are fed a high fat diet (HFD) and become obese, ovarian leptin resistance triggers increased SOCS3 and SMAD7 production, particularly in the TC. Subsequently, SMAD7 inhibits Nodal signalling disrupting its supportive role on steroidogenesis within the TC. Consequently, steroidogenesis is impaired in obese mice.

In summary, this study elucidates the molecular mechanisms compromising ovarian steroidogenesis in obese mothers. We found that increased susceptibility to obesity in mice was associated with systemic hyperleptinemia and hypoestrogenism, the latter mediated by decreased ovarian steroidogenesis, particularly in TC, due to the inhibition of Nodal signalling by SMAD7 (Figure 7). Further studies will unravel how the described signalling pathways might affect oocyte quality and early embryo development.

## CRediT authorship contribution statement

**Karolina Wołodko:** Writing – original draft, Validation, Methodology, Formal analysis, Data curation. **Tjaša Šentjurc:** Methodology, Data curation. **Edyta Walewska:** Methodology, Data curation. **Elżbieta Laniecka:** Formal analysis, Data curation. **Magdalena Jura:** Visualization, Formal analysis, Data curation. **António Galvão:** Writing – review & editing, Supervision, Project administration, Investigation, Formal analysis, Data curation, Conceptualization.

## Disclosure statement

The authors have no conflict of interest to declare.

## Funding sources

Work was supported by grant from the Polish National Centre for Science (No. 2019/34/E/NZ4/00349) awarded to A. G. The costs of Open Access publication were partially covered by the Society for Biology of Reproduction in Poland.

## Declaration of competing interest

All authors of the present work have nothing to declare.

## Data Availability

Data will be made available on request.
