# Supplementary material for: Increased susceptibility to diet-induced obesity in female mice impairs ovarian steroidogenesis: The role of elevated leptin signalling on nodal activity inhibition in theca cells
Source: Mol Metab. 2024 Nov 12;91:102062. doi: 10.1016/j.molmet.2024.102062 (PMC11646782; doi:10.1016/j.molmet.2024.102062)
Supplement: Multimedia component 2 [file mmc2.docx]

| NAME | FORWARD PRIMER | REVERSE PRIMER | TAQMAN PROBE 5’-> 3’ |
| --- | --- | --- | --- |
|  | 5’-> 3’ | 5’-> 3’ | All probes being 5`-6FAM, 3`-TAMRA labelled |
| MEST | GAT CCT ATA AAT CCG TAT CCA GAG TTT T | GGG TAG TGG CTA ATG TGG TCA TC | 6FAM -CCG CGG TCC ACA GTG TCG ATT CT-TAMRA |
| Leptin | AACCCTCATCAAGACCATTGTCA | CCTCTGCTTGGAGGATACC | 6FAM- CAGGATCAATGACATTTCACACACGCAG-TAMRA |
| TNFα | CTGTCTACTGAACTTCGGGGTGAT | CATCAGTTCTATGGCCCAGACC | 6FAM -ATGAGAAGTTCCCAAATGGCCTCCCTC- TAMRA |
| Sfrp5 | CCA AGA TCT GTG CCC AGT GT | 5’-TGC GCA TCT TGA CCA CAA A-3’ | 5’-ATG GCC TCA TGG AAC AGA TGT GCT CC-3’ |

Taqman Gene Expression Assay ID

Cavin Mm00477266_m1

Skp2 Mm00449925_m1
