# Supplementary figures and images for: Increased susceptibility to diet-induced obesity in female mice impairs ovarian steroidogenesis: The role of elevated leptin signalling on nodal activity inhibition in theca cells
Source: Mol Metab. 2024 Nov 12;91:102062. doi: 10.1016/j.molmet.2024.102062 (PMC11646782; doi:10.1016/j.molmet.2024.102062)

A

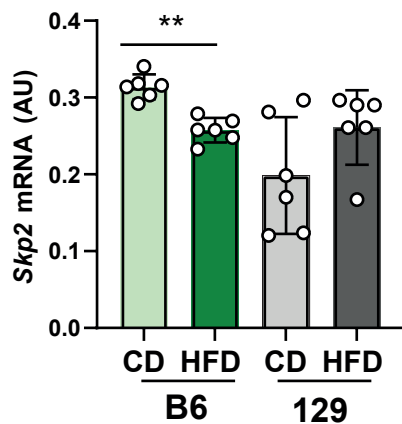

B

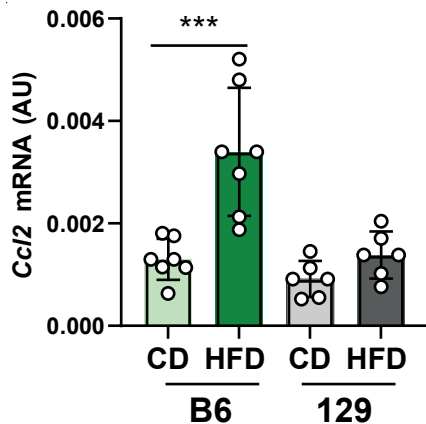

C

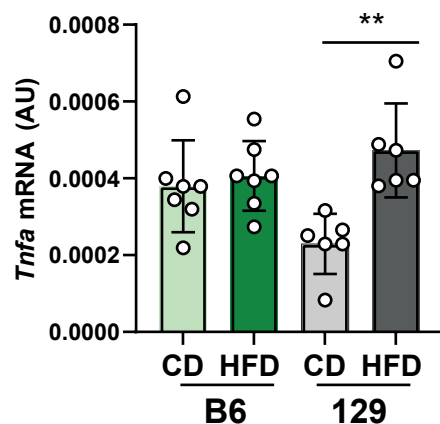

D

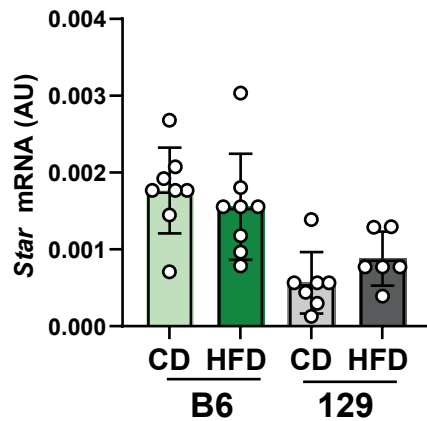

E

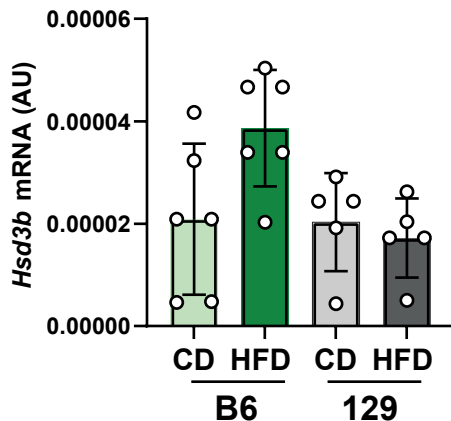

F

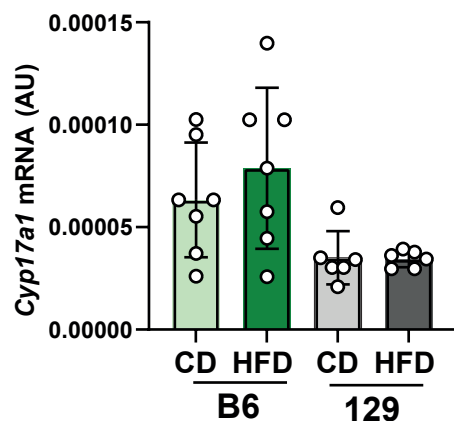

G

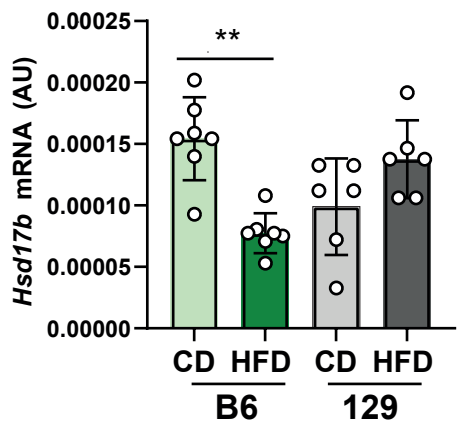

Supplement: Multimedia component 4 [file mmc4.pdf]

A

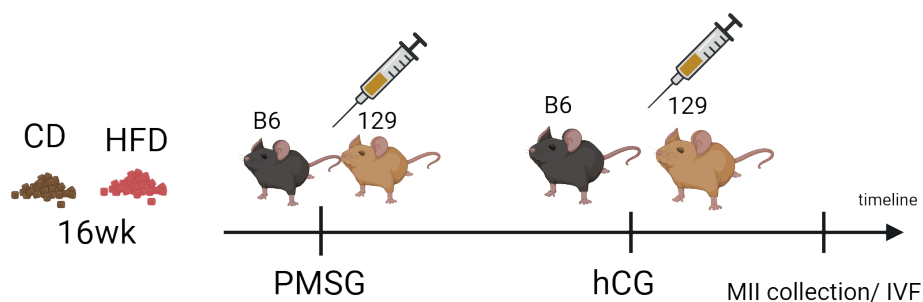

C

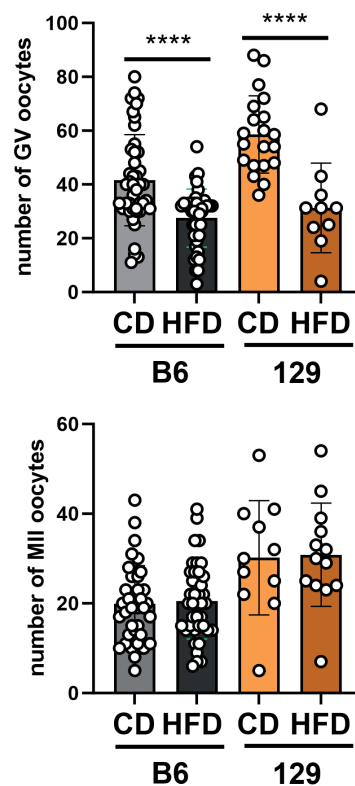

B

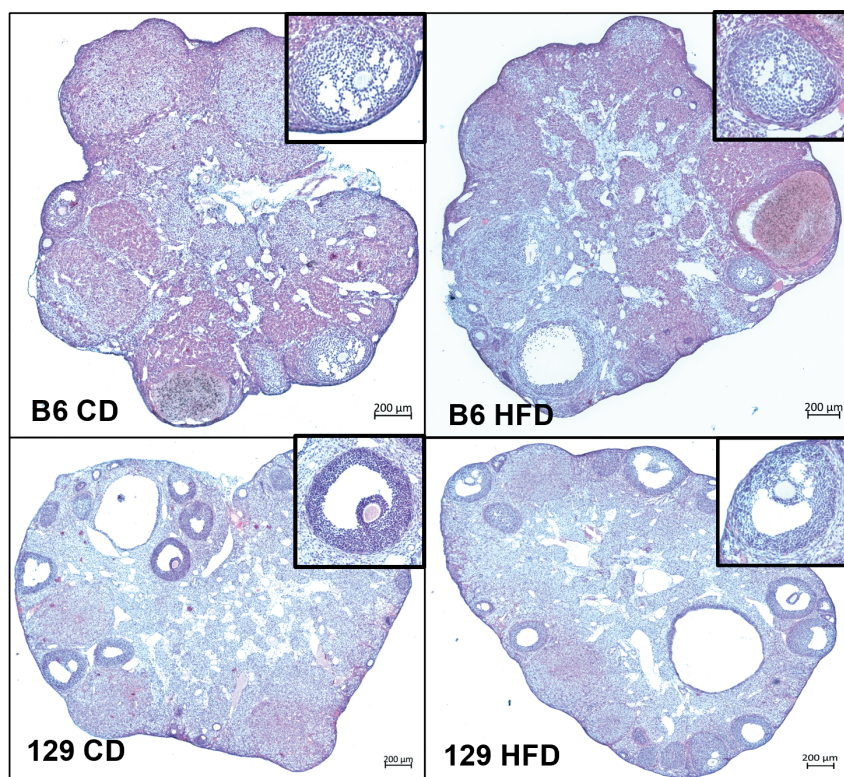

D

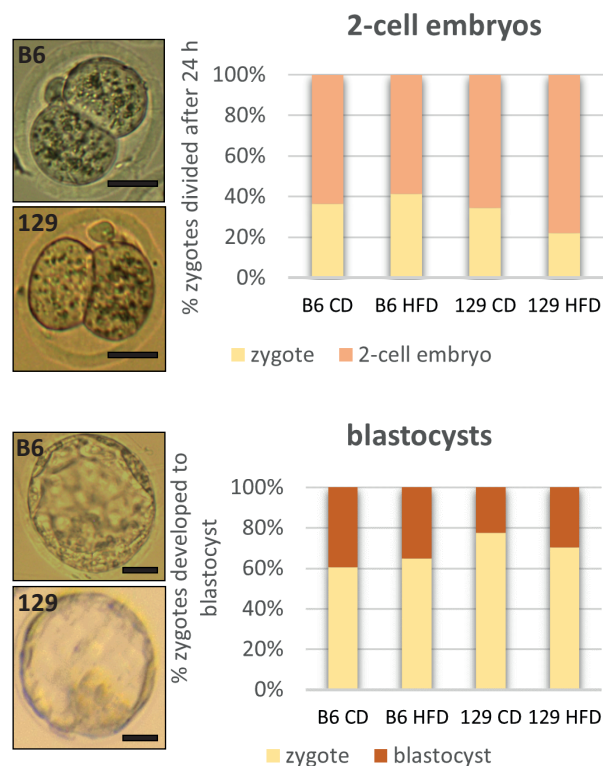

Supplement: Multimedia component 5 [file mmc5.pdf]

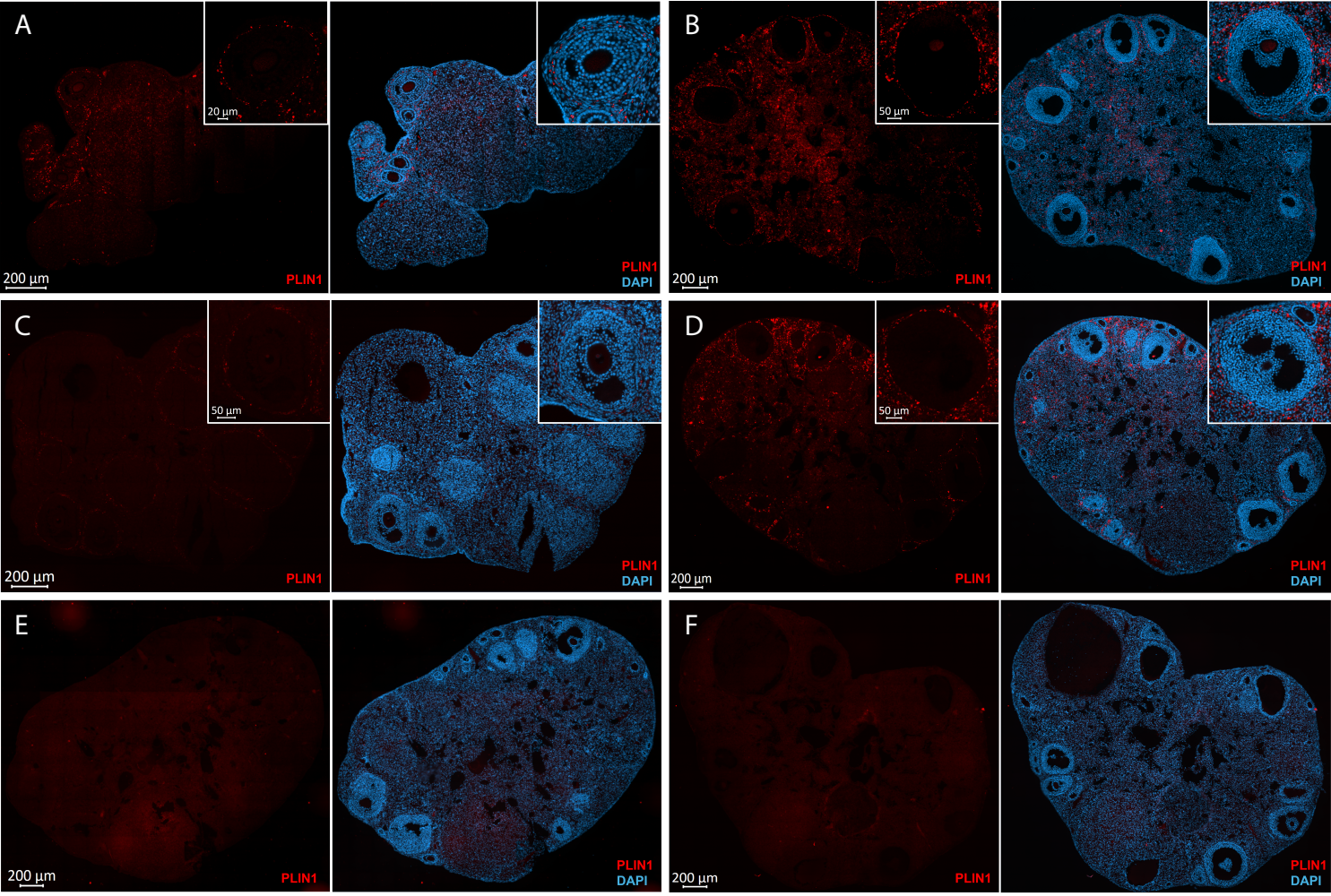

Supplement: Multimedia component 6 [file mmc6.pdf]

**A**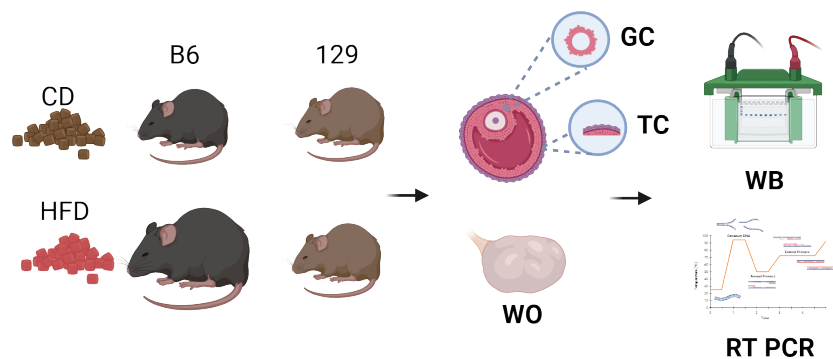**B**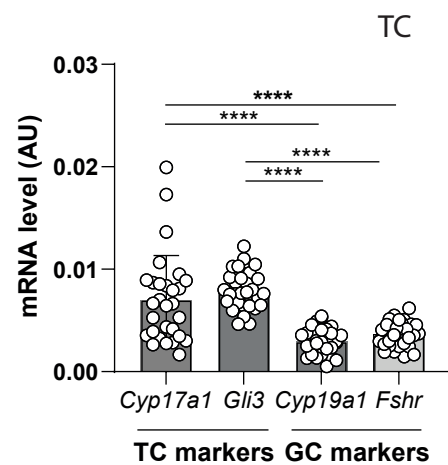**C****GC**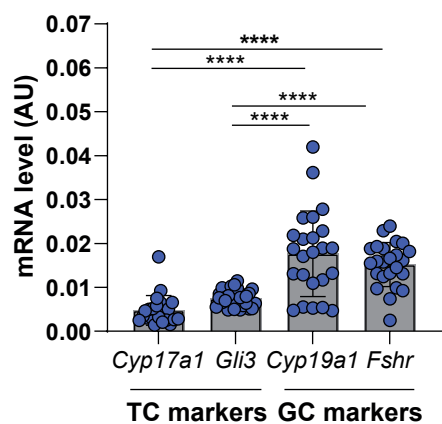**D**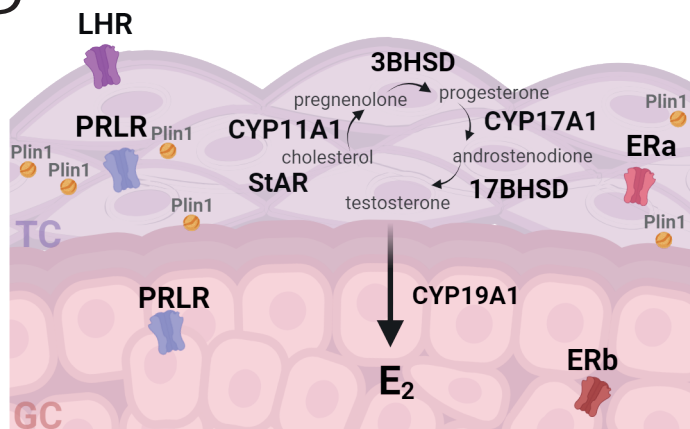**E****WO**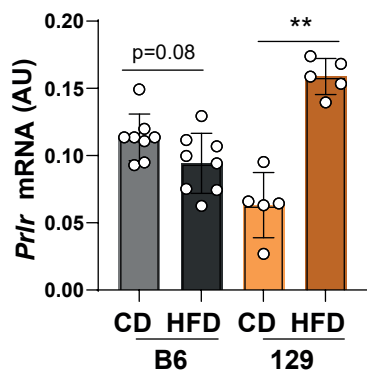**F****WO**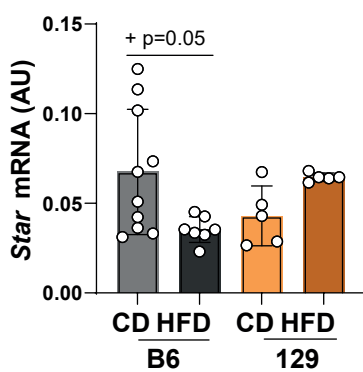**G****GC**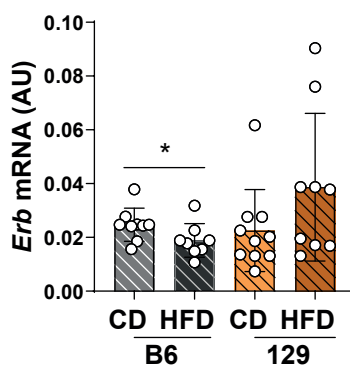**H****WO**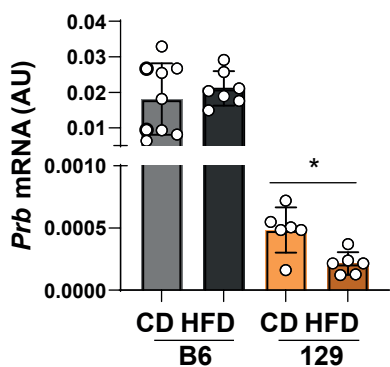**GC**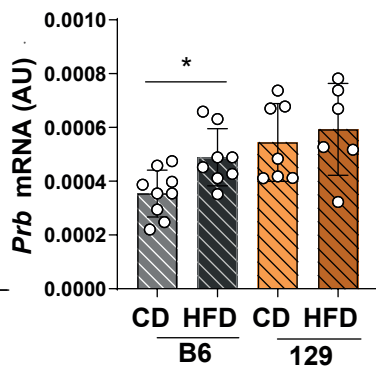**I****TC**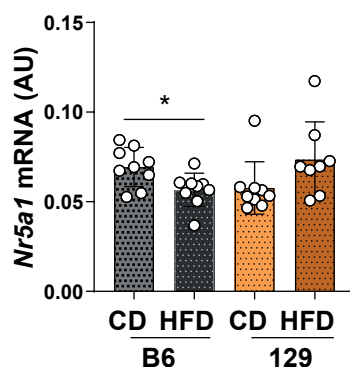**J****TC**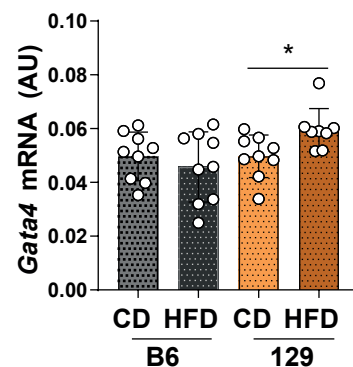

Supplement: Multimedia component 7 [file mmc7.pdf]

A

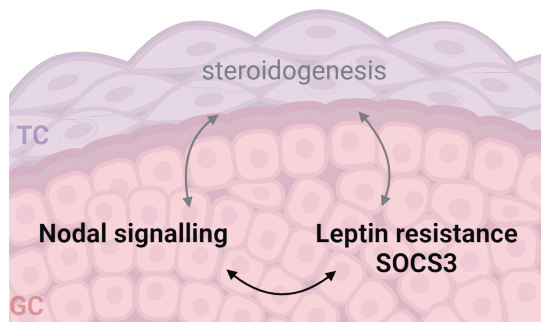

B

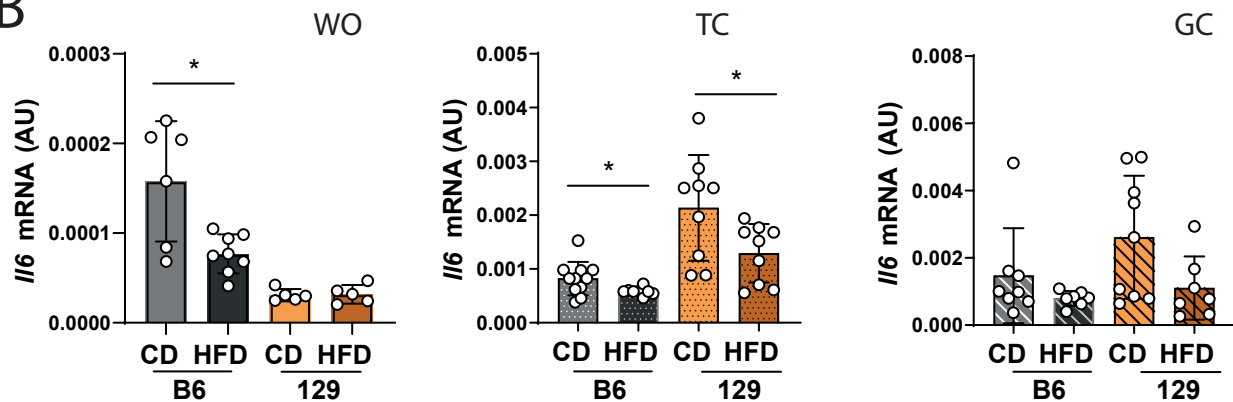

C

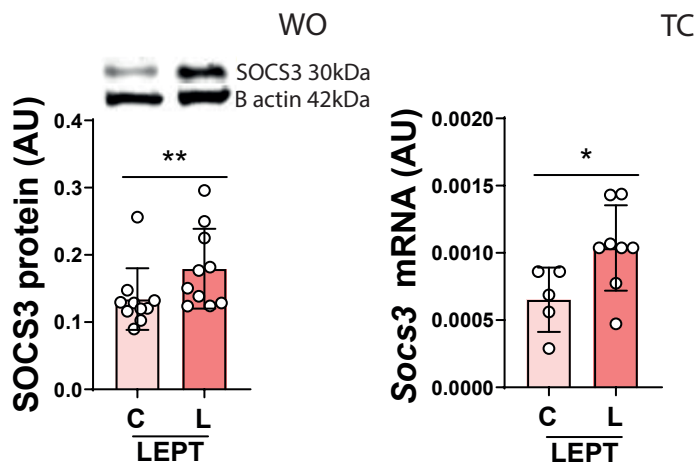

Supplement: Multimedia component 8 [file mmc8.pdf]

A

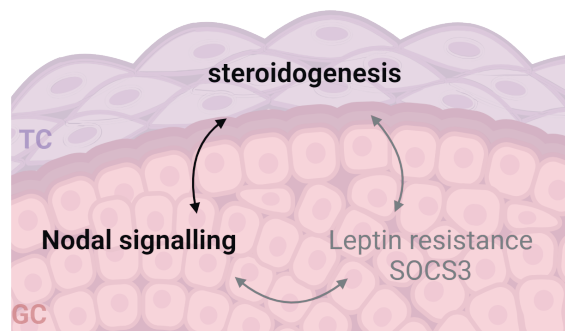

B

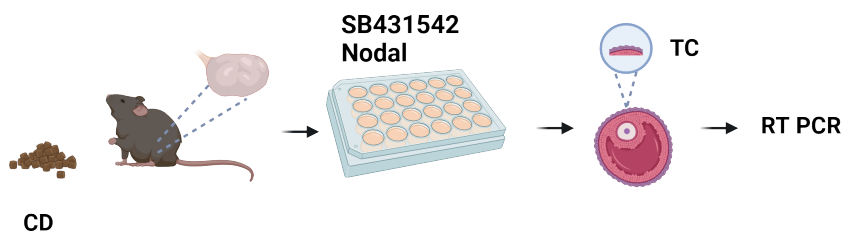

C

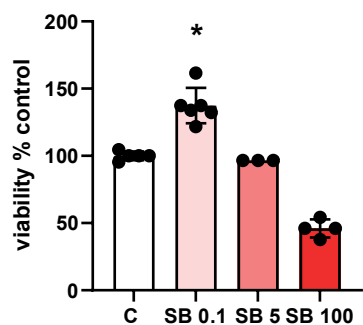

D

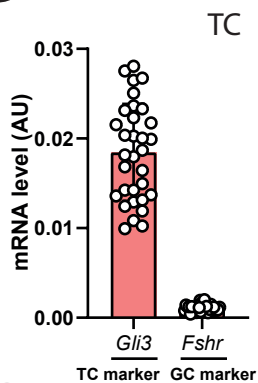

E

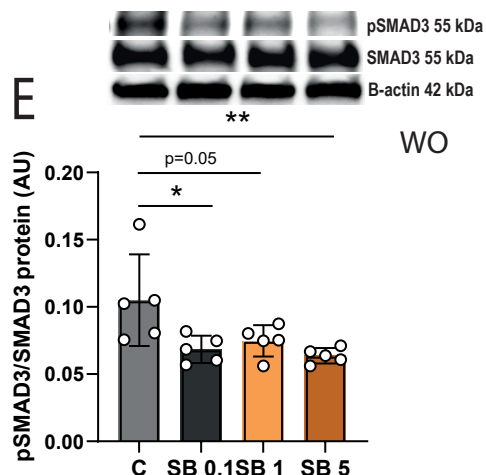

F

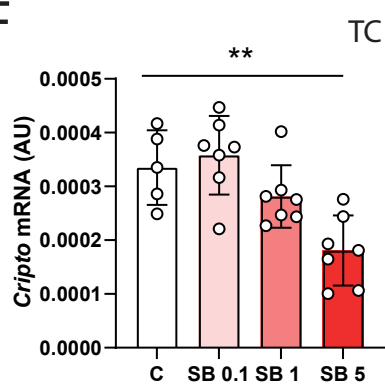

G

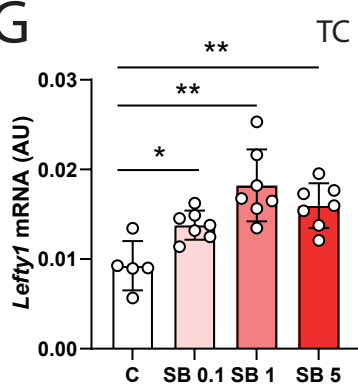

H

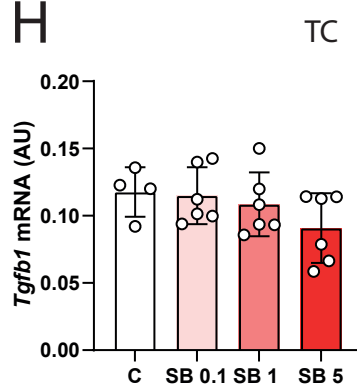

I

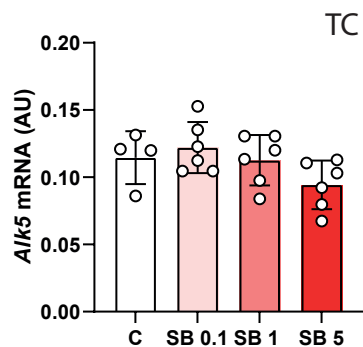

J

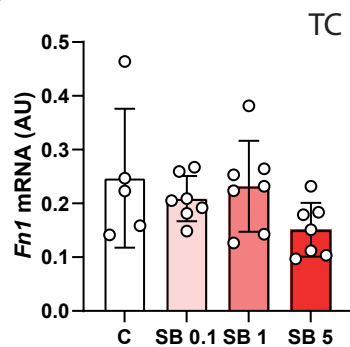

K

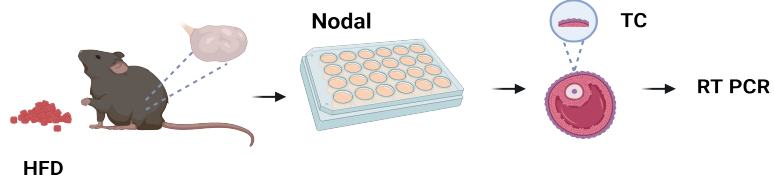

L

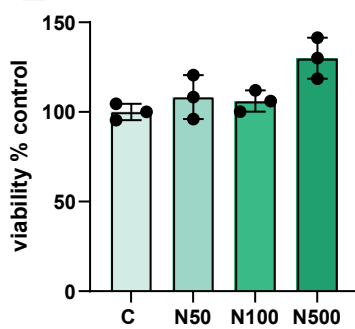

Supplement: Multimedia component 9 [file mmc9.pdf]
